# Responses of alpine summit vegetation under climate change in the transition zone between subtropical and tropical humid environment

**DOI:** 10.1038/s41598-022-17682-2

**Published:** 2022-08-03

**Authors:** Chu-Chia Kuo, Yea-Chen Liu, Yu Su, Ho-Yih Liu, Cheng-Tao Lin

**Affiliations:** 1 Department of Biological Resources, National Chiayi University, No. 300, Shyue-Fu Rd., East District, Chiayi City, 600355 Taiwan; 2 Department of Biological Sciences, National Sun Yat-sen University, No. 70, Lien-Hai Rd., Gushan District, Kaohsiung City, 804201 Taiwan

**Keywords:** Ecology, Community ecology, Plant ecology

## Abstract

Climate change has caused severe impacts on ecosystems and biodiversity globally, especially to vulnerable mountain ecosystems; the summits bear the brunt of such effects. Therefore, six summits in Taiwan were monitored based on a standardized multi-summit approach. We used both statistical downscaling of climate data and vegetation cover data to calculate climate niches to assess the impacts of climate change. Two indicators, thermophilic and moist-philic, were applied to evaluate the overall response of vegetation dynamics. The results revealed that potential evapotranspiration increased significantly and led to a declining tendency in monthly water balance from 2014 to 2019. The general pattern of species richness was a decline. The difference in plant cover among the three surveys showed an inconsistent pattern, although some dominant species expanded, such as the dwarf bamboo *Yushania niitakayamensis.* The thermophilic indicator showed that species composition had changed so that there were more thermophilic species at the three lowest summits. The moist-philization indicator showed a decline of humid-preferred species in the latest monitoring period. Although total precipitation did not decrease, our results suggest that the variability in precipitation with increased temperature and potential evapotranspiration altered alpine vegetation composition and could endanger vulnerable species in the future.

## Introduction

Over the past century, global climate change has resulted in temperature increases, glaciers melting in high mountain areas and polar zones, and even sea level rise, which has modified the distribution range of certain species directly or indirectly^1,2^. Most recent studies suggested that species upward shifting is in response to temperature increase^1,3^, but some studies did not find significant species upward-shifting or they even found in the reverse^4,5^. Several alternative hypotheses are proposed to elucidate species range shifts, such as biotic interactions^6^, increasing nitrogen deposition^7,8^, or water availability^9–13^. Although it is still in debate, the range shifting of species is possibly the most significant effect induced by warming, especially in alpine and mountain regions^2^. The upward range shifts are interpreted commonly as a response to temperature increase, and the alpine plant communities influenced by warming are usually expected to trigger “thermophilization”^2,14–16^, which is a process that describes the decline of cold-adapted species and expansion of warm-adapted species.

Meanwhile, water availability and water-related factors, which include precipitation, snow cover, and soil moisture, also play essential roles in determining the responses of alpine vegetation responses to climate change^9,11,12^. In addition, the alpine environment is a distinct ecosystem that harbors high species richness and endemism^17,18^, in which specialized plant species have adapted to low temperature, short growing seasons, poor nutrient availability, and extreme conditions^19^. Previous studies have also indicated that the plants that inhabit alpine environments are more sensitive to limiting factors, such as temperature and precipitation, than those plants at low elevation^20,21^. Thus, alpine environments are ideal to assess and to monitor the effects of global warming on plant diversity and ecosystem health.

Under the circumstances, the Global Observation Research Initiative in Alpine Environments (GLORIA) was established in the 2000s to collaborate with international scientific communities and to set up a standard multi-summit approach to monitor vegetation changes in high-mountain ecosystems^22^. The target regions of the GLORIA global network also include major biodiversity hotspots of mountain ranges across the world. Most of the monitoring sites were concentrated in temperate and boreal alpine environments, mainly in Europe and America, with only a few in subtropical and tropical regions. The monitoring results of GLORIA sites showed a continental trend of temperature increase during the past few decades^2^. Some studies indicated that the warming rate in winter was remarkably higher than in summer^23,24^. The effects of precipitation-related parameters were also reported^15^, although increasing precipitation and duration of snow cover were usually correlated positively to species richness and plant cover^25^. Therefore, assessing and identifying temperature and precipitation effects on the alpine ecosystem are essential for conservation management, especially in East Asia's humid subtropical and tropical mountains.

East Asia is a plant biodiversity hotspot with high species richness and high endemism^26^. Taiwan is one of the mountainous islands of the East Asian Island Arcs that is located in the transition zone between tropical and subtropical regions, in which there is very high richness in endemic taxa^27^. The backbone mountain range of Taiwan, which includes the Central Mountain Ranges and Hsueh-Shan Mountain Ranges, line in a north–south direction with more than 200 summits higher than 3,000 m. The fragile geology, frequent earthquakes, and humid climate form complex terrestrial habitats. At the same time, the connection with the Eurasian mainland in the past also shaped Taiwan's unique flora; Taiwan has harbored many relic species since the mid-Tertiary in genera, such as *Fagus*, *Taiwania*, and *Trochodendron*^28^. Hence, the alpine summit environments in Taiwan are an excellent field site to monitor the dynamics of these alpine species under climate change. Previous studies showed a general increasing trend in temperature in the high-mountain areas in Taiwan, which resulted in upward shifting of species and modification of the vegetation composition at mountain summits^1,23^. However, most of the studies have aimed at the vegetation responses to temperature, but they have rarely focused on the influences of precipitation or the combined effects of temperature and precipitation.

Meanwhile, the lack of studies about fundamental physiological characteristics of alpine plants in Taiwan also forms a gap in our understanding of vegetation dynamics under climate change. Therefore, we have established standard, long-term vegetation monitoring sites at fifteen summits based on the multi-summit approach of GLORIA since 2008. These monitoring sites were used to monitor and to assess the vegetation responses to climate change, especially the range shifting of alpine species and changes in community composition. Our study used the long-term climatic monitoring data and vegetation composition data of six summits over ten years to construct the climate niche of each species. We tried to address the following questions: 1. What is the dynamic pattern of water resources in the high-mountain summits of Taiwan? 2. What has been the change in vegetation composition, especially for vulnerable species, of the high-mountain summits under climate change? 3. Has thermophilization or moist-philization occurred in alpine summits of Taiwan?

## Results

### Precipitation pattern and water balance

From 2005 to 2019, the annual mean precipitation ranged from 1,848.1 ± 392.2 to 1,999.4 ± 401.7 mm across the three summits in the DAS region and from 2,569.8 ± 750.0 to 3,272.0 ± 1,056.0 mm in the SYU region. The precipitation in both target regions was concentrated from May (late spring) to September (early autumn), which accounted for more than 60% of the precipitation for the year (Fig. S1). There was no significant difference among summits for the three monitoring cycles (2005–2009, 2010–2014, 2015–2019) of annual precipitation (Table S1), and there was no significant linear trend in annual precipitation (Table S2).

**Figure 1:**
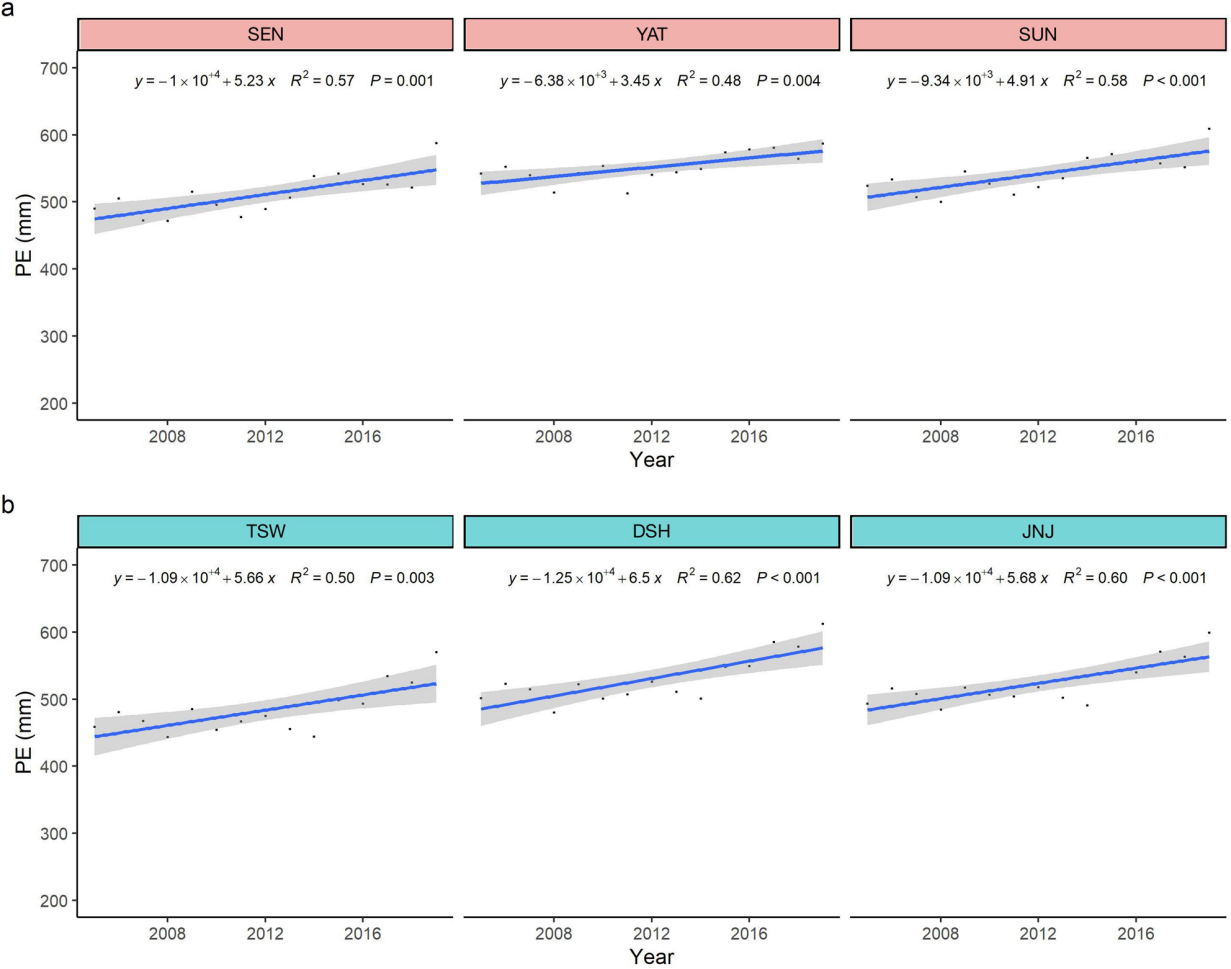
Trends in total annual potential evapotranspiration (PE) among the target summits from 2005 to 2019, (**a**) the Dashueiku Mountain (DAS) region, (**b**) the Syue Mountain (SYU) region.

**Figure 2:**
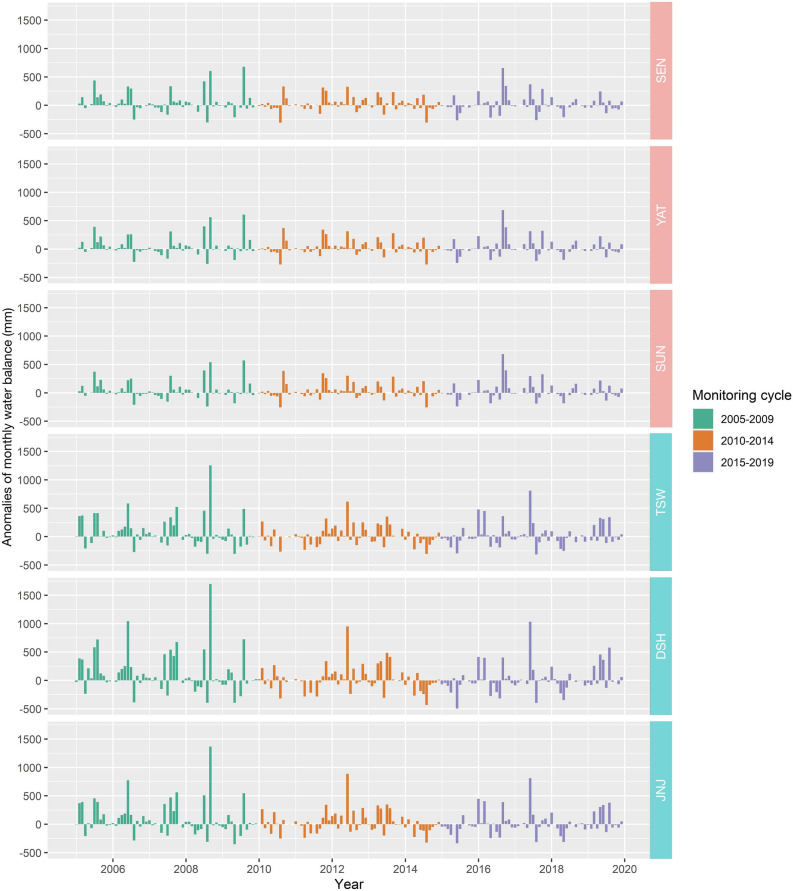
The monthly water balance anomalies in each summit during the monitoring periods from 2005 to 2019. The Dashueiku Mountain (DAS) region included SEN, YAT and SUN summits; the Syue Mountain (SYU) region included TSW, DSH and JNJ.

Although annual precipitation did not decrease during the monitoring cycles, annual potential evapotranspiration increased significantly due to increasing temperatures, and this was followed by a reduction in the water balance (Fig. 1). As a result, the monthly water balance in the third monitoring cycle exhibited a decline, especially during the relative drought season (Fig. S2). Moreover, there were significantly more months with a positive water balance than a negative water balance during the first and second monitoring cycles (Table S3). However, the situation was reversed during the third monitoring cycle; ≥ 50% of the months had a negative water balance (Table S3,  Fig. 2).

### Dynamics of vegetation composition

This study recorded 89 vascular plant species from 32 families during the three monitoring cycles. In addition, there were 44 endemic species, which accounted for 49.4% of all species. There were eleven newly recorded species in the second survey and one species that disappeared in the DAS region, and there were six newly recorded species and two species that disappeared in the SYU region. The number of species generally increased during the second survey (Table S4).

In the third survey, eight new species were recorded, and 21 species had disappeared from the DAS region. In the SYU region, eight new species were recorded, and ten species had disappeared (Table S4). Thus, the number of species declined drastically. However, the number of species that increased or decline was not correlated significantly with mean annual temperature, mean annual precipitation, or the altitude of the summits (Table S5). In the second survey, the colonized species included one nationally endangered species (NEN, *Festuca japonica*) in the DSH, one nationally vulnerable species (NVU, *Dianthus pygmaeus*) in the TSW, and one nationally near threatened species (NNT, *Rubus rolfei*) in the YAT. There were no species listed in the NNT, NVU or NEN categories that disappeared in the second survey. However, four threatened species were absent in the third survey, which included one NEN species (*F. japonica* in the DSH), two NVU species (*Dianthus pygmaeus* in the TSW; *Huperzia selago* in the DSH), and two NNT species (*Berberis kawakamii* in the SUN; *Rubus rolfei* in the TSW).

### Species niche and cover changes

**Figure 3:**
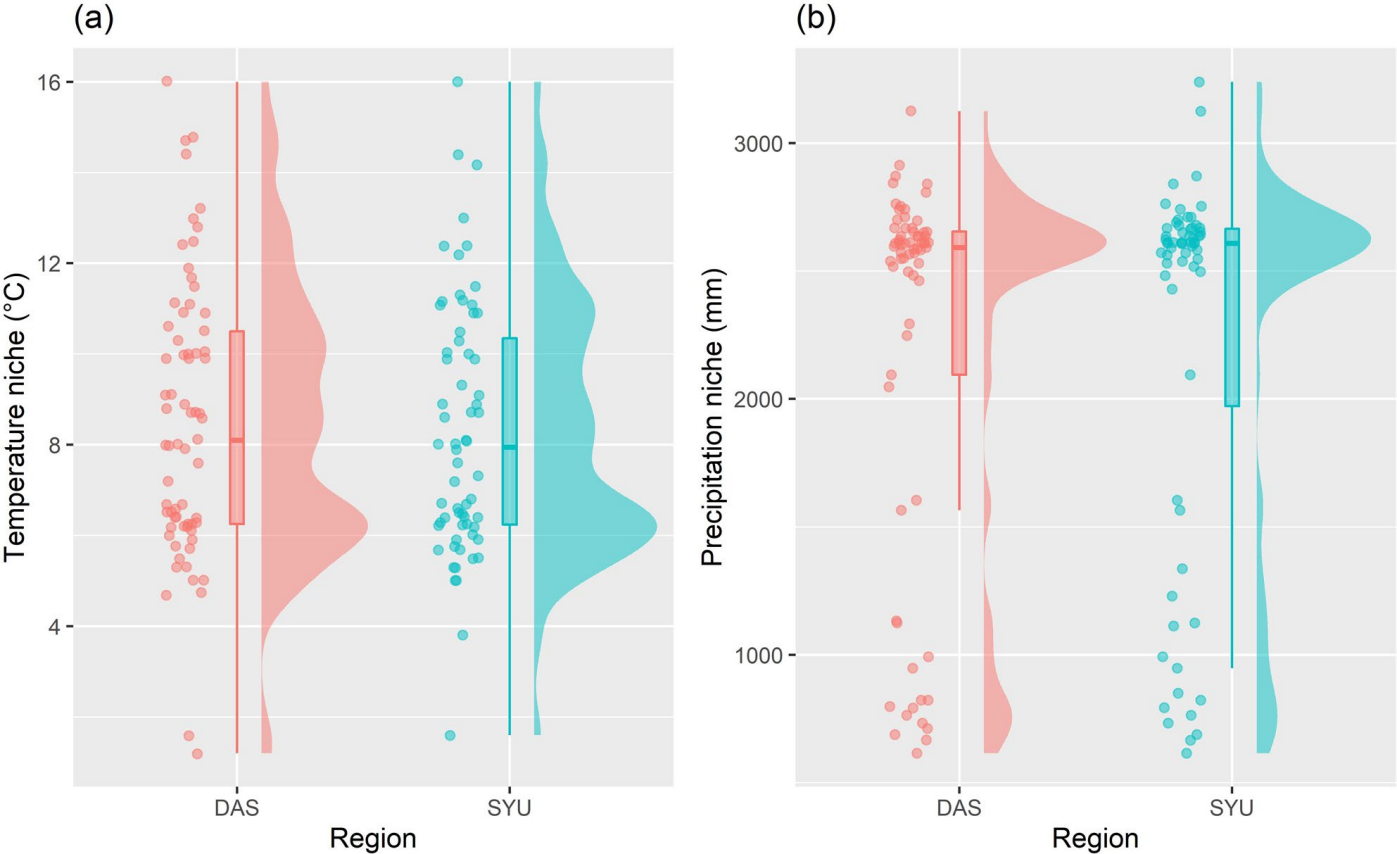
The species climate niche distribution of the Dashueiku Mountain (DAS) and the Syue Mountain (SYU) region, (**a**) temperature niche and (**b**) precipitation niche.

Climate niches and cover changes for species revealed more subtle patterns of variation in plant composition. The temperature and precipitation niches of species in the DAS and SYU regions showed a similar distribution. Most of the species' temperature niches were concentrated between 4–12 °C (Fig. 3a), which approximated the annual average temperature range (7.47–10.34 °C) of summits from 2010 to 2019^23^. Nevertheless, the distribution of the species' precipitation niches was separated into two groups. One of the groups was composed of 61.8% of all species with precipitation niches concentrated from 2,400–2,800 mm. The other groups was composed of 22.5% of all species with precipitation niches that were scattered between 600–1,400 mm (Fig. 3b).

**Figure 4:**
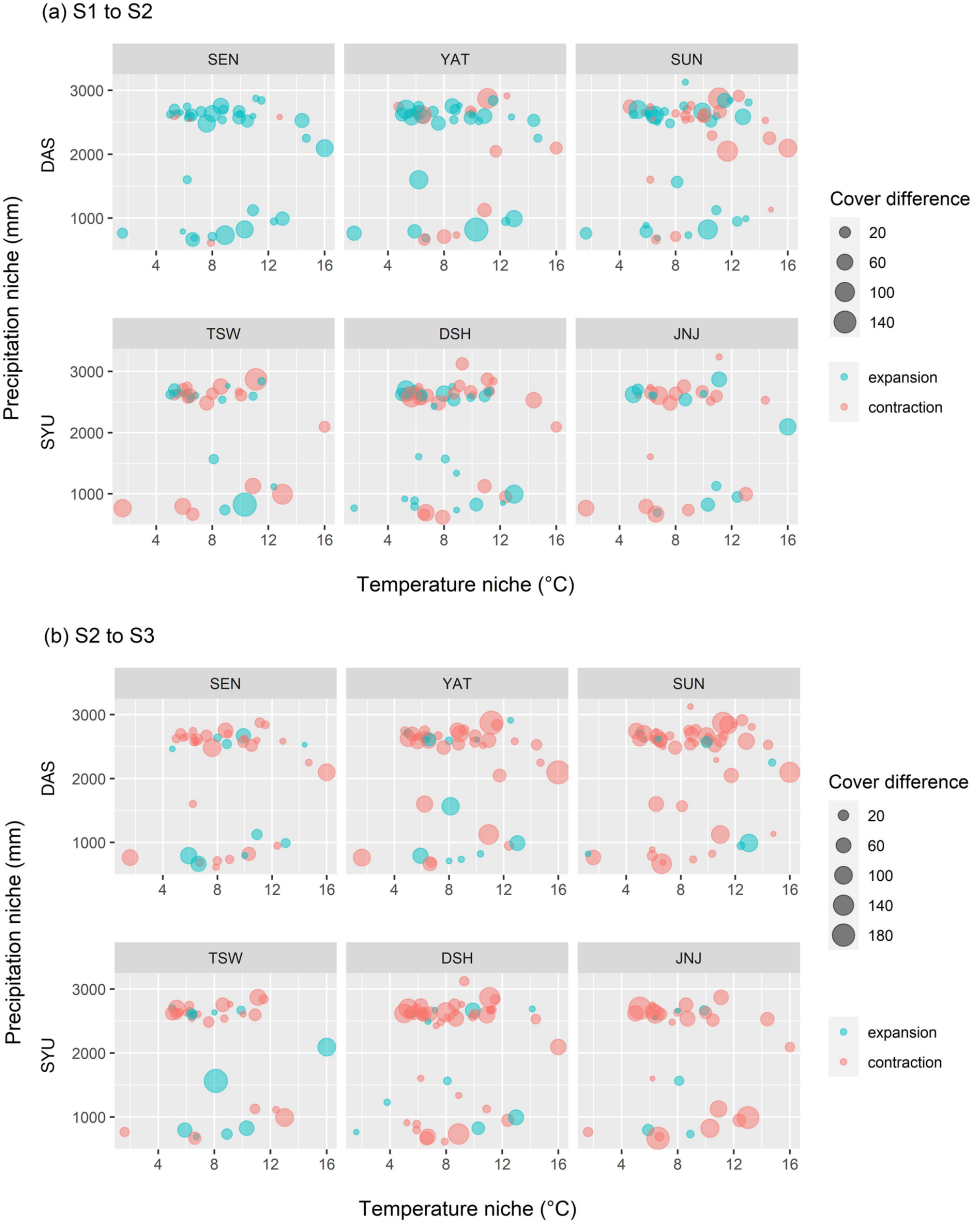
The species climate niche and species cover difference during the three surveys at each summit (summits of the Dashueiku Mountain (DAS) region: SEN, YAT and SEN; the Syue Mountain (SYU) region: TSW, DSH and JNJ), (**a**) from the first survey (S1) to the second (S2), (**b**) from the second survey (S2) to the third (S3).

The climate niche and the cover difference for each species in each summit during the three surveys showed that in the second survey, the cover of most species expanded in the DAS region, and the number of species with cover expansion was not much different from the number of species where cover shrank in the SYU region (Fig. 4a). Some species generally increased their cover at all summits, but their climate niche was inconsistent. Using the climate niche average as the standard (temperature niche average: 8.5 ℃; precipitation niche average: 2,250.5 mm), some species preferred colder and more humid climates, such as *Trisetum spicatum* var. *formosanum*, *Luzula taiwaniana*, and *Veronica morrisonicola*; some preferred drier environments such as *Lycopodium clavatum* and *Lycopodium obscurum,* and some species, such as *Rhododendron rubropilosum* var. *rubropilosum*, *Deschampsia flexuosa*, and endangered species—*Dianthus pygmaeus*, favored more warm and humid climates. The rate of cover change also was not significantly related to the climate niche of species (Table S6).

From the second monitoring cycle to the third, the cover of most species contracted at all summits (Fig. 4b). The generalized linear model also showed that the rate of cover change was significantly negatively related to the precipitation niche (Table S6). More species particularly preferred the humid climate, and the cover was contracted more in the third survey. Some species have decreased drastically among the cover-shrinking species and even disappeared from all the summit plots. Five species listed in the Red List of Vascular Plants of Taiwan disappeared, which included the humid-preferred species, such as *F. japonica*, *D. pygmaeus*, *B. kawakamii*, and *R. rolfei*, and one cryophilic species—*H. selago*. On the other hand, a few species expanded, such as, *Carex oxyandra*, *Lycopodium clavatum*, and *Yushania niitakayamensis*, which exhibited highly competitive growth and dominance.

### The thermophilization and moist-philization of vegetation

**Figure 5:**
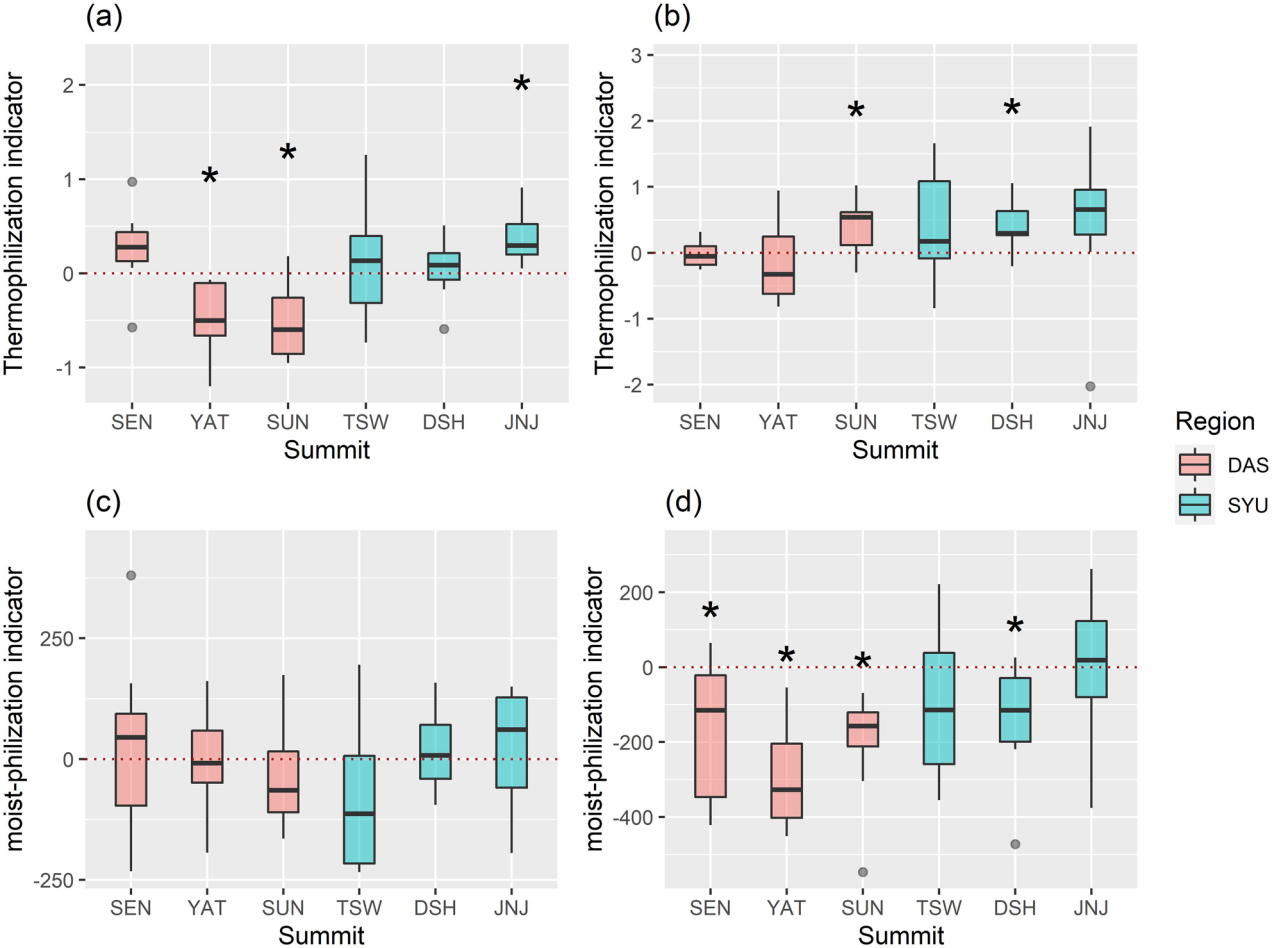
The themophilization and moist-philization indicators of each summit (the Dashueiku Mountain (DAS) region: SEN, YAT and SEN; the Syue Mountain (SYU) region: TSW, DSH and JNJ) during the monitoring periods. (**a**) and (**c**) show the indicators from the first survey to the second; (**b**) and (**d**) show the indicators from the second survey to the third; the star above each summit boxplot means that the indicator value is significantly different from zero in the t-test.

The thermophilization indicator was significantly different from zero at the YAT, SUN, and JNJ summits in the second monitoring cycle (Fig. 5a). Nevertheless, the vegetation only showed a thermophilization trend at the JNJ, in which the thermophilization indicator was significantly more than zero (Fig. 5a, Table S6). In the third monitoring cycle, the thermophilization indicator was significantly greater than zero at the SUN and YAT (Fig. 5b, Table S7). Also, when the outlier was excluded, the indicator of JNJ was significantly over zero (included outliers: t = 1.165, df = 7, *p*-value = 0.282; excluded outliers: t = 3.299, df = 6, p-value = 0.016). Of the six summits surveyed from 2013 to 2020, the three lower summits reflected a significant thermophilic phenomenon.

The moist-philization indicator showed no significant trend at any of the summits from the first to the second monitoring cycle (Fig. 5c). However, the indicator at most summits was significantly below zero in the next monitoring cycle, except at the TSW and JNJ (Table S7). Hence, from 2013 to 2020, the vegetation composition had become dominated by species that preferred a less moist climate (Fig. 5d).

## Discussion

The application of the multi-summit approach in Taiwan^23^ revealed a significant rise in annual mean temperature and winter warming at all summits, but annual precipitation showed no apparent trend. Moreover, we found that the annual precipitation in the study region was abundant from 2005 to 2019. During the monitoring periods, the average annual precipitation of each summit was > 1,500 mm, which was consistent with the characteristics of a humid subtropical climate^29^, and there was no significant decrease over the three monitoring cycles. Therefore, it is difficult to conclude that the summit vegetation was affected by water imbalance based on annual precipitation conditions alone. However, we found that the potential evapotranspiration also increased due to the rise in temperature, which directly reduced the monthly water balance during the third monitoring cycle directly. Because of the increase in potential evapotranspiration and the uneven temporal distribution of precipitation, the monthly negative water balance occurred frequently during the dry season (the fall and winter). In addition, when temperatures were extremely high, water imbalance also occurred during the wet season. Similar situations occurred in 2014–2015, 2018–2019, and 2020–2021 (Fig. 2). Previous research reported that rainfall in Taiwan during the recent 50 years was less than in the past^30^, and the number of typhoons that hit Taiwan directly had decreased in recent years^31^. The rainfall accompanied with typhoon is one of the primary water resources during the wet season in Taiwan^32,33^, and this accounts for nearly 50% of the total annual rainfall^34^; when the typhoons decrease, the water balance during the wet season also declines^32^. However, there is a reduced tendency for typhoons to occur and, therefore, the probability and frequency of drought years might increase in the future^35^.

During the second survey, the number of species generally increased at all the summits, and the cover of numerous species also expanded. Our past study^23^ also showed that the plant diversity index at most of the summits increased slightly. However, even though the overall number of species increased, population sizes of most of the species that had immigrated to the summits were small, which suggested that these immigrants may not have established a stable population yet. Overall, both the immigrant species and the species where the amount of cover changed did not exhibit a consistent pattern in terms of their climate niche. The species with expanding coverage among all summits also had different climate niches. Our results suggested that the difference in species cover might not have been caused by changes in temperature or precipitation directly, but other factors may have been responsible, such as species competition^23,36^, reproduction^37^, or climatic and topographic heterogeneity^38^. Furthermore, the thermophilization indicator at most summits showed no significant changes and did not show a consistent pattern. However, a previous study revealed that the plant diversity has increased slightly and might have been related to winter warming^23^. The effect of winter warming probably facilitated the immigration of species from low elevations, but it did not affect the cold-adapted species as of yet^4^. Although the high-mountain vegetation was temperature-sensitive^39^, the changes in vegetation might still result from abiotic drivers, biotic interactions, and spatial- and temporal-scale issues, which could lead to nonlinear responses^40^.

In the third monitoring cycle, a significant increase in average annual temperature and winter temperature, and the decline of species number and diversity index generally occurred at most of the summits^23^. Furthermore, the water balance was often lower than the long-term average, and a negative water balance was occurred frequently, combined with the rising temperature and potential evapotranspiration. As a result, the cover of most species that preferred moist environments, declined and disappeared. In addition, linear regression analysis revealed that the higher the precipitation niche, the higher was the rate of decline in species cover. The thermophilization indicator and moist-philization indicator (Fig. 5) showed that the vegetation composition was structured by species that preferred lower precipitation and warmer climate, which indicated that the vegetation was affected by the decrease in water balance and rising temperature^25,41^.

Water resources were limited in the summit areas because there was no topographic runoff and no groundwater layer, and precipitation was probably the only water source^42^. The lack of developed soil layer created a low capacity for water storage in alpine environments^42^. Therefore, the combining of the declines in precipitation and increased temperatures often caused drought events in alpine areas^10^. Water deficit forces plants to trigger the drought-resistant mechanisms, which include a reduction in leaf number, diminished growth, wilting of the aboveground parts, enhanced dry matter allocation to the roots, or a shift into the reproductive stage^43,44^. Hence, plants might survive after a drought, but the drought tends to result in smaller plant size and reduced total coverage. Frequent or intense droughts might also cause plant death and affect seed dormancy, germination, and establishment, and conclusively bring on a decline in the plant population^25^. In addition to the water imbalance events, the competition caused by the expansion of *Y. niitakayamensis* might further lead to a decline in other species cover decrease or even their disappearance. The dense culm and robust rhizome of *Y. niitakayamensis* occupied most of the light and soil resources, which made it difficult for other species to become established^45,46^. Furthermore, with the increased temperatures, *Y. niitakayamensis* which was limited originally by low temperatures, extended its range gradually and crowded out other plants. This resulted in a decreased diversity^47^ and occasional water imbalance events that might accelerate this process^48^.

The study was implemented with the specifications of the GLORIA field manual to reduce the interference of other unknown factors. The investigation time occurred during the growing season in alpine environments to minimize observer bias. But resurveys often involve sources of unwanted variability^49^. Because the GLORIA protocol was designed to detect long-term trends in alpine vegetation and the global warming effect, the resurvey was implemented every 5–10 years. During the monitoring cycle, extreme and local meteorological events might cause fluctuations in vegetation composition, but these were difficult to detect with a quinquennial resurvey frequency^40^. In addition, although we resurveyed in the same season as the previous survey, there was still a possibility that the plant phenological stage was delayed by water shortage in the spring^50,51^. Of course, this delay might cause a bias that led us to believe that some species were scarce or even disappeared since the last survey.

Nevertheless, our study still provides some crucial information. Because there was a ~ 100 km distance between the DAS and the SYU regions, and we resurveyed in the different years, the general decrease in plants that preferred moisture may not have been caused by a single or local meteorological event, but something that occurred generally throughout the high mountains of Taiwan. Approximately, nearly 50% of the species in the target summits were endemic, which indicated the particularity of the alpine vegetation in Taiwan. However, such particularity might be threatened indirectly by extreme climate events, increased presence of competitors, or habitat degradation^23,52^. Furthermore, previous studies had reported that the plant productivity declined or vegetation diversity was lost, due to drought events in alpine environments^25,41,53,54^. Still, those studies were mainly in a specific region with a precipitation regime that produced pronounced dry and wet seasons. This study found that even in areas where the annual rainfall was ≥ 1500 mm, water imbalance events still occurred due to temporally uneven precipitation and increased potential evapotranspiration caused by rising temperatures. It extended the plant growth season^23^, expanded the *Y. niitakayamensis* population, and accelerated soil water loss^36,48^. Those factors may increase the probability of drought events, threaten the species in the Red List and endemic species, and alter composition of the alpine vegetation.

## Material and methods

### Study area

**Figure 6:**
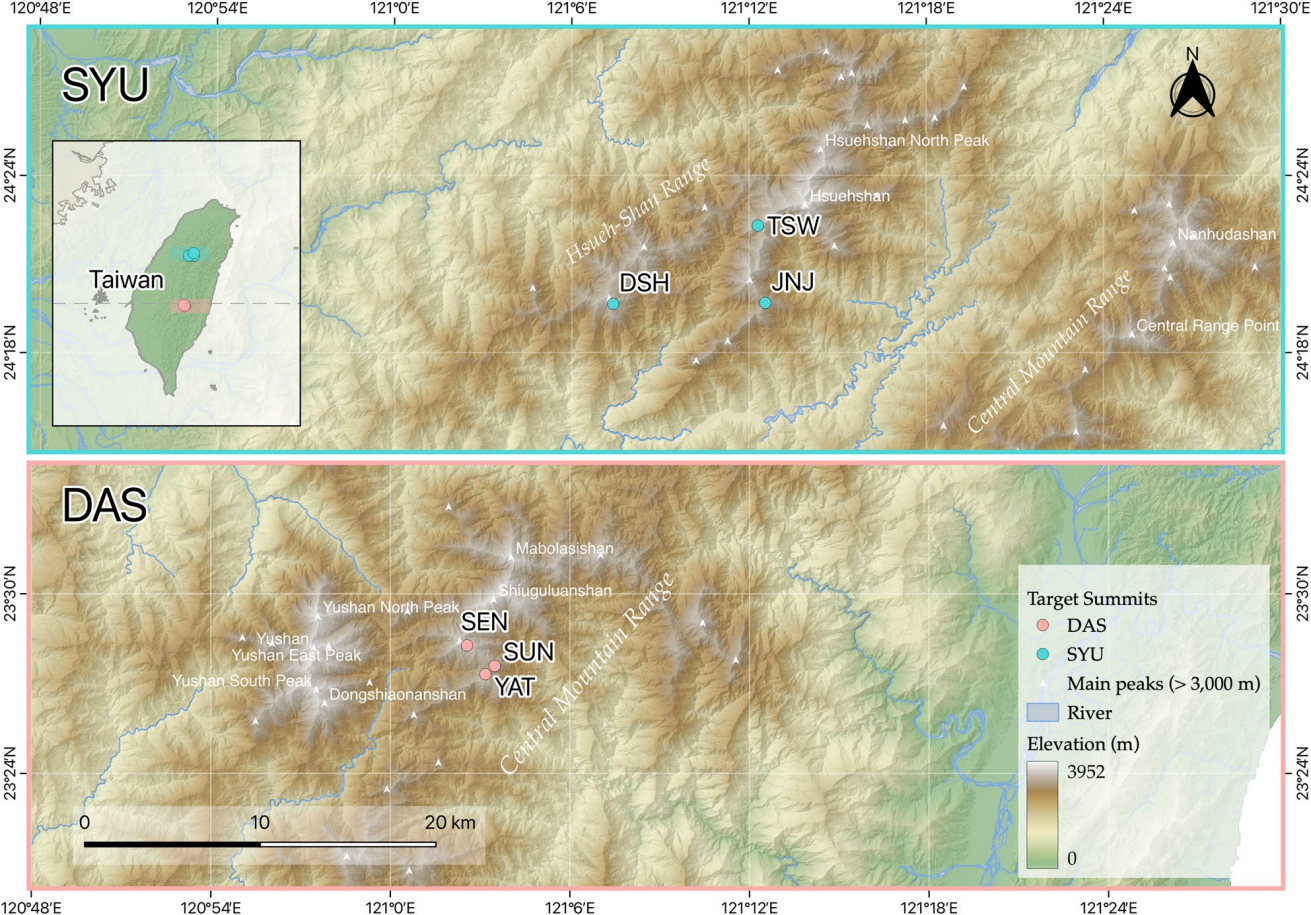
The location of long-term monitoring summits in alpine environments of Taiwan. The summits of Dashueiku Mountain (DAS) region: SEN, YAT and SEN; the Syue Mountain (SYU) region: TSW, DSH and JNJ). The map was drawn by QGIS (version 3.24; URL: https://qgis.org).

The study area was located in Taiwan and consists mainly of two mountain regions (Fig. 1), the Dashueiku Mountain region (DAS) and the Syue Mountain region (SYU). The DAS region was in the middle of the Central Mountain Range, and three monitored summits were selected (SEN, SUN, and YAT) with elevations that ranged from 3,255–3,610 m a.s.l. From 2010–2021, the mean annual temperature was 9.24°C^23^, the mean annual precipitation was 1915.7 mm, and the main growing season (monthly mean temperature > 5 °C) was from April (March in YAT) to November. The SYU region was northwest of the Central Mountain Range, and it included the TSW, DSH, and JNJ monitored summits, with a mean annual temperature of 8.44°C^23^, mean annual precipitation of 2684.6 mm, and the main growing season was from April to November (from May to October in TSW). The DAS and SYU regions were located in Yushan National Park and Shei-Pa National Park, respectively, and both were biodiversity hotspots in Taiwan^55^. The six summits were selected according to the criteria of the GLORIA field manual^22,23^ and were distributed from the lower alpine to the alpine zone (Table 1, Fig. 6). The rainfall in the two regions is mainly controlled by the monsoons and typhoons^56^, with relatively low rainfall in winter due to the northeastern monsoon, rainfall in spring due to the monsoon front, and heavy rainfall in summer due to the southwestern monsoons and typhoons^57^.

**Table 1:** The basic information for all summits used as study areas in our survey of vegetation in the mountains of Taiwan (adapted from Kuo et al^23^).

Summit Code	Longitude	Latitude	Initial date	Elevation (m)	Vegetation zone
TW-DAS-SUN	121° 03′ 29.2″	23°27′35.5″	2008-09-05	3.255	Lower alpine
TW-DAS-YAT	121° 03′ 10.7″	23°27′19.0″	2008-09-10	3.363	Lower alpine
TW-DAS-SEN	121° 02′ 33.9″	23°28′16.6″	2008-09-17	3.610	Alpine
TW-SYU-JNJ	121° 12′ 33.0″	24°19′41.0″	2009-06-25	3.299	Lower alpine
TW-SYU-DSH	121° 07′ 24.0″	24°19′39.0″	2009-09-25	3.509	Lower alpine
TW-SYU-TSW	121° 12′ 18.0″	24°22′18.0″	2009-06-16	3.524	Lower alpine

### Vegetation monitoring

**Figure 7:**
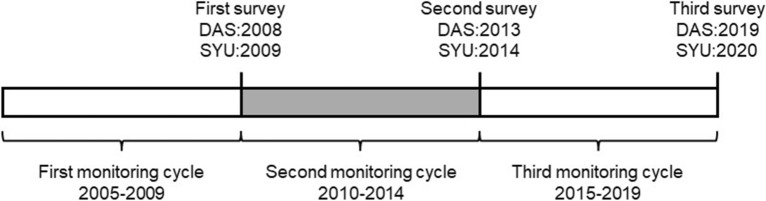
The definition of each survey year and the monitoring cycles in this study.

From 2008 to 2020, all six summits were surveyed three times. We defined the survey interval period as the monitoring cycle as a unit of climate data analysis (Fig. 7)**.** The first monitoring cycle was five years before the first survey. The first survey was conducted in the DAS area in the summer of 2008, and the second and third surveys were conducted in the summers of 2013 and 2019, respectively. The sample areas were set up and surveyed by a standard procedure using the GLORIA multi-summit approach^22^.

Each summit was divided into eight summit area sections, which included upper and lower sections in the four cardinal directions (N, E, S, W). The upper section was five meters vertically below the highest summit point (HSP); the lower section was between 5 and 10 m vertically below the HSP. The top surface cover for each summit area section was estimated by percentage. All of the vascular plants and their abundances were estimated visually. Plant abundance was recorded by the following classes: r! = very rare, r = rare, s = scattered, c = common, or d = dominant.

### Weather data and water balance

The interpolated daily weather data at 1-km resolution from the Taiwan Climate Change Projection Information Platform (TCCIP, URL: https://tccip.ncdr.nat.gov.tw) were used to represent the precipitation of each summit, and then we calculated the annual and monthly precipitation from 2005 to 2019. ANOVA analysis was used to compare precipitation among the three monitoring cycles, and linear regression was used to examine whether there was a linear trend in precipitation over the years. To detect the anomalies of water balance under climate change, we used both precipitation data and the potential evapotranspiration based on Thornthwaite’s method^58^. The Thornthwaite’s method can be used when only temperature data are available, and it was computed using Eq. (1):

1 $$PET = 1.6b\left[ \frac{10t}{I} \right]^{a}$$

where PET = potential evapotranspiration; b = (total monthly daylight hours)/360; t = the mean monthly air temperature (℃); I = the annual heat index;

$$a = 0.49239 + 0.0179I - 0.000077I^{2} + 0.000000675I^{3}$$

We also calculated the monthly mean temperature from TCCIP data and computed the monthly potential evapotranspiration of each summit to ensure data consistency. The monthly anomalies of water balance were defined as the monthly water balance minus the median value of the month from 2005 to 2019, and the monthly water balance was the difference between monthly precipitation and potential evapotranspiration. We compared whether the number of positive values (i.e., number of the monthly precipitation was higher than potential evapotranspiration) was significantly higher or lower than negative values during the monitoring cycles among each summit by using a Wilcoxon signed rank test.

### Species climate niche

Due to the lack of basic physiological information on alpine species in Taiwan, it was difficult to determine the habitat preferred by each species. Using open-source databases to retrieve the known distributional locations of species to construct the species climate niche has been adopted in many studies^14,59–61^. This study used the species records from the Global Biodiversity Information Facility (GBIF, URL: https://www.gbif.org) to build the species temperature and precipitation niches (i.e., the optimum temperature and precipitation of species).

First, we retrieved all records for each species from the GBIF database and excluded species records without spatial information (Table S8). Then, the records with completely overlapping points were excluded. After filtering the data, we used the species coordinates to extract mean annual temperature and mean annual precipitation of species habitat from the global statistical downscaling climate database—CHELSA (Climatologies at high resolution for the earth’s and surface areas; URL: https://www.chelsa-climate.org)^62^. The climate factors were long-term averages from 1979 to 2013, and the resolution was 30 arc-sec (about 800 m in Taiwan). After obtaining the climate factors for each species, the median annual mean temperature and annual precipitation were used, which reduce the effect of extreme values, as the representative values for each species temperature and precipitation niches.

### Variation in species cover

The plant abundance using the Braun-Blanquet scale was converted to numerical values in the following classes: r! = 1, r = 5, s = 25, c = 50, and d = 75. Because the area of each section’s plot was different, and the vascular plant total coverage varied in each survey, we multiplied each plant abundance and total coverage to obtain the actual cover of each species in each section’s plot. We then summed them to get the total cover of each species at the summit. Also, the cover difference between the two surveys was calculated from Eq. (2):

2 $$C_{d} = C_{t} \cdot TC_{t} - C_{t - 1} \cdot TC_{t - 1}$$

where $C_{t}$ is the species cover value of the section in the survey *t;* $TC_{t}$ is the vascular plant total coverage (%) of the section in survey t, and *t-1* is the previous survey.

Because the cover differences were distributed non-normally and to allow species with different cover classes to be compared at the same scale^63^, we used the rate of change in species cover to represent the cover variation between two surveys. This rate was calculated with the following Eq. (3):

3 $$CCR = \frac{{C_{t} \cdot TC_{t} + 1}}{{C_{t - 1} \cdot TC_{t - 1} + 1}}$$

where *CCR* is rate of change in species cover, $C_{t}$ is the species cover value of the section in survey *t*; $TC_{t}$ is the vascular plant total coverage (%) of the section in survey t, and *t* − 1 refers to the previous survey.

The generalized linear model (GLM) was employed to test the relationship between climate niche and species cover change rate. We used gamma distribution with logarithm link function in GLM. The species cover change rate was used as the response variable, and the temperature niche and precipitation niche were the independent variables.

### The thermophilization and moist-philization indicator

We used the thermophilization and moist-philization indicator to quantify the effect of climate change on the composition of alpine vegetation. Those indicators were derived from previous studies^2,14,15^. First, the vegetation thermic and moist indicators of each summit section and survey were calculated as the community-weighted mean of the species niche that was weighed by species cover. Subsequently, the thermophilization and moist-philization indicators, as to the shift over time, were obtained by subtracting the post-survey thermic (or moist) indicator from the pre-survey indicator. The formulas are represented by Eqs. (4) and (5):

4 $$CW_{t} = \frac{{\sum C_{i} \cdot N_{i} }}{{\sum C_{i} }}$$

5 $$VT = CW_{t} - CW_{t - 1}$$

where $CW_{t}$ is the vegetation community weight of in the specific climatic factor at survey *t* (temperature or precipitation), $C_{i}$ is the cover of species *i*; $N_{i}$ is the climate niche of species *I*, $VT$ is the vegetation trend indicator (thermophilization or moist-philization), and *t-1* refers to the previous survey. The *t*-test was used to test whether the thermophilization and moist-philization indicators at each summit differed significantly from zero.

All the calculations were completed in R 4.0.3^64^ and by using the following packages: SPEI for potential evapotranspiration function^65^, and rgbif for obtaining occurrences of plants from GBIF^66^.

## Supplementary Information

Supplementary Information.

## Data Availability

The meteorological and climate data used in this study are accessible through TCCIP (https://tccip.ncdr.nat.gov.tw) and the CHELSA website (https://chelsa-climate.org). Species occurrences can be retrieved from the GBIF website (https://gbif.org). The plant survey data are available from the corresponding author upon reasonable request.

## References

[1] Chen IC, Hill JK, Ohlemuller R, Roy DB, Thomas CD (2011). Rapid range shifts of species associated with high levels of climate warming. Science.

[2] Gottfried M (2012). Continent-wide response of mountain vegetation to climate change. Nat. Clim. Change.

[3] Rumpf SB (2018). Range dynamics of mountain plants decrease with elevation. Proc. Natl. Acad. Sci..

[4] Gigauri K, Akhalkatsi M, Abdaladze O, Nakhutsrishvili G (2016). Alpine plant distribution and thermic vegetation indicator on GLORIA summits in the Central Greater Caucasus. Pak. J. Bot..

[5] Gritsch A, Dirnböck T, Dullinger S (2016). Recent changes in alpine vegetation differ among plant communities. J. Veg. Sci..

[6] Speed JDM, Austrheim G, Hester AJ, Mysterud A (2012). Elevational advance of alpine plant communities is buffered by herbivory. J. Veg. Sci..

[7] Grytnes JA (2014). Identifying the driving factors behind observed elevational range shifts on European mountains. Global Ecol. Biogeogr..

[8] Johnson DR, Ebert-May D, Webber PJ, Tweedie CE (2011). Forecasting alpine vegetation change using repeat sampling and a novel modeling approach. Ambio.

[9] Amagai Y, Kudo G, Sato K (2018). Changes in alpine plant communities under climate change: Dynamics of snow-meadow vegetation in northern Japan over the last 40 years. Appl. Veg. Sci..

[10] Crimmins SM, Dobrowski SZ, Greenberg JA, Abatzoglou JT, Mynsberge AR (2011). Changes in climatic water balance drive downhill shifts in plant species’ optimum elevations. Science.

[11] Engler R (2011). 21st century climate change threatens mountain flora unequally across Europe. Global Change Biol..

[12] Matteodo M, Ammann K, Verrecchia EP, Vittoz P (2016). Snowbeds are more affected than other subalpine–alpine plant communities by climate change in the Swiss Alps. Ecol. Evol..

[13] Tingley MW, Monahan WB, Beissinger SR, Moritz C (2009). Birds track their Grinnellian niche through a century of climate change. Proc. Natl. Acad. Sci..

[14] Cuesta F (2020). Thermal niche traits of high alpine plant species and communities across the tropical Andes and their vulnerability to global warming. J. Biogeogr..

[15] Hamid M, Khuroo AA, Malik AH, Ahmad R, Singh CP (2020). Assessment of alpine summit flora in Kashmir Himalaya and its implications for long-term monitoring of climate change impacts. J. Mt. Sci..

[16] Steinbauer K, Lamprecht A, Semenchuk P, Winkler M, Pauli H (2019). Dieback and expansions: Species-specific responses during 20 years of amplified warming in the high Alps. Alpine Bot..

[17] Noroozi J (2018). Hotspots within a global biodiversity hotspot-areas of endemism are associated with high mountain ranges. Sci. Rep..

[18] Testolin R (2021). Global patterns and drivers of alpine plant species richness. Global Ecol. Biogeogr..

[19] Körner, C. in *Alpine Plant Life* Ch. 1. Plant ecology at high elevations, 1–22 (Springer, 2021).

[20] Smith JG, Sconiers W, Spasojevic MJ, Ashton IW, Suding KN (2012). Phenological changes in alpine plants in response to increased snowpack, temperature, and nitrogen. Arct. Antarct. Alp. Res..

[21] Körner, C. *Alpine Plant Life*. (Springer, 2021).

[22] Pauli, H. *et al. The GLORIA field manual–standard Multi-Summit approach, supplementary methods and extra approaches*. 5th edn, (GLORIA-Coordination, Austrian Academy of Sciences & University of Natural Resources and Life Sciences, 2015).

[23] Kuo C-C, Su Y, Liu H-Y, Lin C-T (2021). Assessment of climate change effects on alpine summit vegetation in the transition of tropical to subtropical humid climate. Plant Ecol..

[24] Suonan J, Classen AT, Zhang Z, He JS (2017). Asymmetric winter warming advanced plant phenology to a greater extent than symmetric warming in an alpine meadow. Funct. Ecol..

[25] Lamprecht A (2021). Changes in plant diversity in a water-limited and isolated high-mountain range (Sierra Nevada, Spain). Alpine Bot..

[26] Barthlott W, Mutke J, Rafiqpoor D, Kier G, Kreft H (2005). Global centers of vascular plant diversity. Nova Acta Leopold..

[27] Kier G (2009). A global assessment of endemism and species richness across island and mainland regions. Proc. Natl. Acad. Sci..

[28] Huang S-F (2011). Historical biogeography of the flora of Taiwan. J. Natl. Taiwan Museum.

[29] Beck HE (2018). Present and future Köppen-Geiger climate classification maps at 1-km resolution. Sci. Data.

[30] TCCIP. The past and future of climate in Taiwan. 1–31 (National Science and Technology Center for Disaster Reduction & Research Center for Environmental Change, Academia Sinica, New Taipei City, 2018).

[31] Central Weather Bureau. in *The Typhoon Database* (ed Central Weather Bureau;) (https://rdc28.cwb.gov.tw/TDB/, 2021).

[32] Henny L, Thorncroft CD, Hsu H-H, Bosart LF (2021). Extreme rainfall in Taiwan: Seasonal statistics and trends. J. Climate.

[33] Tu J-Y, Chou C (2013). Changes in precipitation frequency and intensity in the vicinity of Taiwan: Typhoon versus non-typhoon events. Environ. Res. Lett..

[34] Liang A, Oey L, Huang S, Chou S (2017). Long-term trends of typhoon-induced rainfall over Taiwan: In situ evidence of poleward shift of typhoons in western North Pacific in recent decades. J. Geophys. Res. Atmos..

[35] Lee Y-C, Wang C-C, Weng S-P, Chen C-T, Cheng C-T (2019). Future projections of meteorological drought characteristics in Taiwan. Atmos. Sci..

[36] Kudo G, Kawai Y, Amagai Y, Winkler DE (2017). Degradation and recovery of an alpine plant community: Experimental removal of an encroaching dwarf bamboo. Alpine Bot..

[37] Richman SK, Levine JM, Stefan L, Johnson CA (2020). Asynchronous range shifts drive alpine plant–pollinator interactions and reduce plant fitness. Global Change Biol..

[38] Spasojevic MJ, Bowman WD, Humphries HC, Seastedt TR, Suding KN (2013). Changes in alpine vegetation over 21 years: Are patterns across a heterogeneous landscape consistent with predictions?. Ecosphere.

[39] Rogora M (2018). Assessment of climate change effects on mountain ecosystems through a cross-site analysis in the Alps and Apennines. Sci. Total Environ..

[40] Malanson GP, Resler LM, Butler DR, Fagre DB (2019). Mountain plant communities: Uncertain sentinels?. Prog. Phys. Geogr. Earth Environ..

[41] Berauer BJ (2019). Low resistance of montane and alpine grasslands to abrupt changes in temperature and precipitation regimes. Arct Antarct. Alp. Res..

[42] Körner, C. in *Alpine Plant Life* Ch. 9. Water relations, 333–383 (Springer, 2021).

[43] Cai Y (2015). Photosynthetic response of an alpine plant, rhododendron delavayi Franch, to water stress and recovery: The role of Mesophyll conductance. Front. Plant Sci..

[44] Farooq, M., Wahid, A., Kobayashi, N., Fujita, D. & Basra, S. M. A. in *Sustainable Agriculture* (eds E. Lichtfouse *et al.*) 153–188 (Springer, 2009).

[45] Greenwood S, Chen JC, Chen CT, Jump AS (2015). Temperature and sheltering determine patterns of seedling establishment in an advancing subtropical treeline. J. Veg. Sci..

[46] Morley PJ, Donoghue DNM, Chen JC, Jump AS (2020). Montane forest expansion at high elevations drives rapid reduction in non-forest area, despite no change in mean forest elevation. J. Biogeogr..

[47] Salick J, Ghimire SK, Fang Z, Dema S, Konchar KM (2014). Himalayan alpine vegetation, climate change and mitigation. J. Ethnobiol..

[48] Winkler M (2016). The rich sides of mountain summits–a pan-European view on aspect preferences of alpine plants. J. Biogeogr..

[49] Verheyen K (2016). Combining biodiversity resurveys across regions to advance global change research. Bioscience.

[50] Ganjurjav H (2016). Complex responses of spring vegetation growth to climate in a moisture-limited alpine meadow. Sci. Rep..

[51] Nagy L, Kreyling J, Gellesch E, Beierkuhnlein C, Jentsch A (2013). Recurring weather extremes alter the flowering phenology of two common temperate shrubs. Int. J. Biometeorol..

[52] Jump AS, Huang T-J, Chou C-H (2012). Rapid altitudinal migration of mountain plants in Taiwan and its implications for high altitude biodiversity. Ecography.

[53] Cowles J, Boldgiv B, Liancourt P, Petraitis PS, Casper BB (2018). Effects of increased temperature on plant communities depend on landscape location and precipitation. Ecol. Evol..

[54] Oldfather MF, Ackerly DD (2019). Increases in thermophilus plants in an arid alpine community in response to experimental warming. Arct. Antarct. Alp. Res..

[55] Shao K-T (2012). Taiwan’s biodiversity research achievements over the past 10 years (2001–2011). Biodivers. Sci..

[56] Chen J-M, Lu F-C, Kuo S-L, Shih C-F (2005). Summer climate variability in Taiwan and associated large-scale processes. J. Meteorol. Soc. Japan.

[57] Chen T-C, Wang S-Y, Huang W-R, Yen M-C (2004). Variation of the East Asian summer monsoon rainfall. J. Climate.

[58] Thornthwaite CW (1948). An approach toward a rational classification of climate. Geogr. Rev..

[59] Kambach S (2019). Of niches and distributions: Range size increases with niche breadth both globally and regionally but regional estimates poorly relate to global estimates. Ecography.

[60] Luna B, Moreno JM (2010). Range-size, local abundance and germination niche-breadth in Mediterranean plants of two life-forms. Plant Ecol..

[61] Newbold T (2010). Applications and limitations of museum data for conservation and ecology, with particular attention to species distribution models. Prog. Phys. Geog..

[62] Karger DN, Wilson AM, Mahony C, Zimmermann NE, Jetz W (2021). Global daily 1 km land surface precipitation based on cloud cover-informed downscaling. Sci. Data.

[63] Welham, S. J., Gezan, S. A., Clark, S. J. & Mead, A. *Statistical Methods in Biology: Design and Analysis of Experiments and Regression*. (Chapman and Hall/CRC, 2014).

[64] R: A Language and Environment for Statistical Computing v. 4.0.3 (2021).

[65] Beguería S, Vicente-Serrano SM, Reig F, Latorre B (2014). Standardized precipitation evapotranspiration index (SPEI) revisited: Parameter fitting, evapotranspiration models, tools, datasets and drought monitoring. Int. J. Climatol..

[66] rgbif: Interface to the Global Biodiversity Information Facility API v. 3.7.1 (2022).

