# Supplementary material for: Responses of alpine summit vegetation under climate change in the transition zone between subtropical and tropical humid environment
Source: Sci Rep. 2022 Aug 3;12:13352. doi: 10.1038/s41598-022-17682-2 (PMC9349258; doi:10.1038/s41598-022-17682-2)
Supplement: Supplementary file 1 — Supplementary Information. [file 41598_2022_17682_MOESM1_ESM.docx]

Supplementary materials

**Table S1.** The ANOVA analysis result of annual precipitation of each summit among the three monitoring cycles.

| **Summit** | **F statistic** | ***p*-value** | **df** |
| --- | --- | --- | --- |
| SEN | 0.841 | 0.455 | 2 |
| YAT | 0.327 | 0.728 | 2 |
| SUN | 0.175 | 0.842 | 2 |
| TSW | 1.162 | 0.346 | 2 |
| DSH | 2.584 | 0.117 | 2 |
| JNJ | 1.512 | 0.260 | 2 |

**Table S2**. The linear regression result for annual precipitation and years at each summit.

| **Summit** | **R^2^** | **Adj. R^2^** | **sigma** | **F** | ***p*-value** | **df** |
| --- | --- | --- | --- | --- | --- | --- |
| SEN | 0.099 | 0.030 | 395.702 | 1.429 | 0.253 | 1 |
| YAT | 0.045 | -0.028 | 402.064 | 0.617 | 0.446 | 1 |
| SUN | 0.026 | -0.049 | 401.633 | 0.348 | 0.566 | 1 |
| TSW | 0.112 | 0.043 | 733.590 | 1.635 | 0.223 | 1 |
| DSH | 0.221 | 0.161 | 967.096 | 3.692 | 0.077 | 1 |
| JNJ | 0.189 | 0.126 | 777.316 | 3.026 | 0.106 | 1 |

**Table S3**. The Wilcoxon signed rank test with continuity correction between the months in which positive water balance and negative water balance during the three monitoring cycles.

| **Monitoring cycle** | **Alternative** | **V-value** | ***p*-value** |
| --- | --- | --- | --- |
| 1 | greater | 21 | 0.018 |
| 2 | greater | 15 | 0.028 |
| 3 | less | 0 | 0.018 |

**Table S4**. The checklist of vascular plants and their differences during the three surveys

| **Species** | **DAS** | | | | | **SYU** | | | | |
| --- | --- | --- | --- | --- | --- | --- | --- | --- | --- | --- |
|  | **S1^1^** | **S2^1^** | **S3^1^** | **Diff**  **(1, 2) ^2^** | **Diff**  **(2, 3) ^2^** | **S1** | **S2** | **S3** | **Diff**  **(1, 2)** | **Diff**  **(2, 3)** |
| *Abies kawakamii* (Hayata) T.Itô |  |  |  |  |  | 1 | 1 | 1 |  |  |
| *Agrostis infirma* Buse var. *infirma* | 1 | 1 |  |  | − | 1 | 1 | 1 |  |  |
| *Ainsliaea latifolia* (D.Don) Sch.Bip. subsp. *henryi* (Diels) H.Koyama |  |  |  |  |  | 1 | 1 | 1 |  |  |
| *Amitostigma alpestre* Fukuy. |  | 1 |  | + | − |  |  |  |  |  |
| *Anaphalis morrisonicola* Hayata | 1 | 1 | 1 |  |  |  |  |  |  |  |
| *Anaphalis nepalensis* (Spreng.) Hand.-Mazz. | 1 | 1 | 1 |  |  | 1 | 1 | 1 |  |  |
| *Arabidopsis lyrata* (L.) O’Kane & Al-Shehbaz subsp. *kamchatica* (Fisch. ex DC.) Hultén |  |  |  |  |  |  |  | 1 |  | + |
| *Arenaria takasagomontana* (Masam.) S.S.Ying |  |  |  |  |  |  | 1 |  | + | − |
| *Artemisia kawakamii* Hayata |  | 1 |  | + | − |  |  |  |  |  |
| *Athyrium reflexipinnum* Hayata |  | 1 |  | + | − |  |  | 1 |  | + |
| *Berberis kawakamii* Hayata | 1 | 1 |  |  | − |  |  |  |  |  |
| *Berberis morrisonensis* Hayata | 1 |  | 1 | − | + | 1 | 1 | 1 |  |  |
| *Boschniakia himalaica* Hook.f. & Thomson | 1 | 1 |  |  | − | 1 | 1 | 1 |  |  |
| *Brachypodium kawakamii* Hayata | 1 | 1 | 1 |  |  |  |  | 1 |  | + |
| *Carex brachyanthera* Ohwi |  |  |  |  |  | 1 | 1 |  |  | − |
| *Carex breviculmis* R. Br. |  | 1 | 1 | + |  | 1 | 1 | 1 |  |  |
| *Carex chrysolepis* Franch. & Sav. |  | 1 |  | + | − | 1 | 1 |  |  | − |
| *Carex filicina* Nees |  |  |  |  |  |  |  | 1 |  | + |
| *Carex orthostemon* Hayata | 1 | 1 |  |  | − |  |  |  |  |  |
| *Carex oxyandra* Kudo |  | 1 | 1 | + |  |  | 1 | 1 | + |  |
| *Carex tristachya* var. *pocilliformis* (Boott) Kük. | 1 | 1 | 1 |  |  | 1 | 1 | 1 |  |  |
| *Cirsium arisanense* Kitam. | 1 | 1 |  |  | − | 1 | 1 |  |  | − |
| *Cirsium kawakamii* Hayata |  |  | 1 |  | + |  |  | 1 |  | + |
| *Clematis montana* Buch.-Ham. ex DC. |  |  |  |  |  | 1 | 1 | 1 |  |  |
| *Dendrolycopodium juniperoideum* (Sw.) A.Haines | 1 | 1 |  |  | − | 1 | 1 | 1 |  |  |
| *Deschampsia flexuosa* (L.) Trin. | 1 | 1 | 1 |  |  | 1 | 1 | 1 |  |  |
| *Deyeuxia suizanensis* (Hayata) Ohwi | 1 | 1 | 1 |  |  |  |  |  |  |  |
| *Dianthus pygmaeus* Hayata | 1 | 1 | 1 |  |  | 1 | 1 | 1 |  |  |
| *Diphasiastrum multispicatum* (J.H.Wilce) Holub | 1 | 1 | 1 |  |  |  |  |  |  |  |
| *Diphasiastrum veitchii* (Christ) Holub | 1 | 1 |  |  | − | 1 | 1 | 1 |  |  |
| *Dryopteris alpestris* Tagawa | 1 | 1 |  |  | − | 1 | 1 | 1 |  |  |
| *Dryopteris expansa* (C.Presl) Fraser-Jenk. & Jermy |  | 1 |  | + | − |  | 1 |  | + | − |
| *Elymus formosanus* (Honda) Á.Löve | 1 | 1 | 1 |  |  | 1 | 1 | 1 |  |  |
| *Euphrasia transmorrisonensis* Hayata var. *transmorrisonensis* | 1 | 1 | 1 |  |  |  |  |  |  |  |
| *Festuca japonica* Makino |  |  |  |  |  |  | 1 |  | + | − |
| *Festuca ovina* L. | 1 | 1 | 1 |  |  | 1 | 1 | 1 |  |  |
| *Festuca rubra* L. | 1 | 1 | 1 |  |  |  |  |  |  |  |
| *Gaultheria itoana* Hayata | 1 | 1 | 1 |  |  | 1 | 1 | 1 |  |  |
| *Gentiana arisanensis* Hayata | 1 | 1 | 1 |  |  | 1 | 1 | 1 |  |  |
| *Gentiana davidii* var. *formosana* (Hayata) T.N.Ho | 1 | 1 | 1 |  |  | 1 | 1 | 1 |  |  |
| *Gentiana flavomaculata* Hayata var. *flavomaculata* |  |  |  |  |  | 1 |  |  | − |  |
| *Gentiana scabrida* Hayata | 1 | 1 | 1 |  |  |  |  | 1 |  | + |
| *Geranium hayatanum* Ohwi |  |  |  |  |  |  |  | 1 |  | + |
| *Gnaphalium involucratum* var. *simplex* DC. | 1 | 1 | 1 |  |  |  |  |  |  |  |
| *Helictotrichon abietetorum* (Ohwi) Ohwi | 1 | 1 |  |  | − | 1 | 1 | 1 |  |  |
| *Hieracium morii* Hayata | 1 | 1 | 1 |  |  | 1 | 1 | 1 |  |  |
| *Huperzia selago* (L.) Bernh. ex Schrank & Mart. |  |  |  |  |  |  | 1 |  | + | − |
| *Hydrocotyle setulosa* Hayata |  | 1 |  | + | − |  |  |  |  |  |
| *Hypericum nagasawae* Hayata | 1 | 1 | 1 |  |  | 1 | 1 | 1 |  |  |
| *Juniperus formosana* Hayata var. *formosana* | 1 | 1 | 1 |  |  | 1 | 1 | 1 |  |  |
| *Juniperus squamata* Lamb. | 1 | 1 | 1 |  |  | 1 | 1 | 1 |  |  |
| *Leontopodium microphyllum* Hayata |  |  |  |  |  | 1 | 1 | 1 |  |  |
| *Luzula effusa* Buchenau |  |  |  |  |  | 1 | 1 |  |  | − |
| *Luzula taiwaniana* Satake | 1 | 1 | 1 |  |  | 1 | 1 | 1 |  |  |
| *Lycopodium clavatum* L. | 1 | 1 | 1 |  |  | 1 | 1 | 1 |  |  |
| *Maianthemum formosanum* (Hayata) LaFrankie |  |  |  |  |  | 1 | 1 | 1 |  |  |
| *Miscanthus sinensis* Andersson | 1 | 1 | 1 |  |  | 1 | 1 | 1 |  |  |
| *Myriactis humilis* Merr. |  |  |  |  |  | 1 | 1 | 1 |  |  |
| *Origanum vulgare* L. |  |  | 1 |  | + |  |  |  |  |  |
| *Phleum alpinum* L. |  |  | 1 |  | + |  |  |  |  |  |
| *Picris hieracioides* subsp. *morrisonensis* (Hayata) Kitam. |  |  | 1 |  | + |  |  | 1 |  | + |
| *Pieris taiwanensis* Hayata | 1 | 1 | 1 |  |  |  |  |  |  |  |
| *Pinus armandii* var. *mastersiana* (Hayata) Hayata | 1 | 1 |  |  | − | 1 | 1 |  |  | − |
| *Pinus taiwanensis* Hayata | 1 | 1 | 1 |  |  |  |  |  |  |  |
| *Platanthera brevicalcarata* Hayata |  |  | 1 |  | + | 1 | 1 | 1 |  |  |
| *Polygala japonica* Houtt. | 1 | 1 | 1 |  |  |  |  |  |  |  |
| *Potentilla leuconota* D.Don | 1 | 1 |  |  | − | 1 | 1 | 1 |  |  |
| *Potentilla tugitakensis* Masam. |  |  | 1 |  | + |  |  |  |  |  |
| *Rhododendron pseudochrysanthum* Hayata | 1 | 1 | 1 |  |  | 1 | 1 | 1 |  |  |
| *Rhododendron rubropilosum* Hayata var. *rubropilosum* | 1 | 1 | 1 |  |  | 1 | 1 | 1 |  |  |
| *Ribes formosanum* Hayata |  |  |  |  |  | 1 | 1 |  |  | − |
| *Rosa transmorrisonensis* Hayata | 1 | 1 | 1 |  |  |  |  |  |  |  |
| *Rubus rolfei* S. Vidal | 1 | 1 | 1 |  |  | 1 | 1 | 1 |  |  |
| *Salix taiwanalpina* var. *takasagoalpina* (Koidz.) S.S.Ying |  | 1 |  | + | − |  |  |  |  |  |
| Hayata | 1 | 1 | 1 |  |  |  |  |  |  |  |
| *Sedum morrisonense* Hayata |  |  |  |  |  | 1 | 1 | 1 |  |  |
| *Selliguea quasidivaricata* (Hayata) H.Ohashi & K.Ohashi |  | 1 |  | + | − |  |  |  |  |  |
| *Senecio morrisonensis* Hayata |  |  |  |  |  |  | 1 | 1 | + |  |
| *Solidago virgaurea* var. *leiocarpa* Miq. | 1 | 1 | 1 |  |  | 1 | 1 | 1 |  |  |
| *Spiraea formosana* Hayata |  | 1 |  | + | − | 1 | 1 | 1 |  |  |
| *Spiraea hayatana* H.L. Li |  |  | 1 |  | + |  |  |  |  |  |
| *Spiraea morrisonicola* Hayata | 1 | 1 |  |  | − | 1 |  |  | − |  |
| *Swertia macrosperma* (C.B.Clarke) C.B.Clarke | 1 | 1 | 1 |  |  |  |  |  |  |  |
| *Trichophorum subcapitatum* (Thwaites & Hook.) D.A. Simpson | 1 | 1 | 1 |  |  | 1 | 1 | 1 |  |  |
| *Trisetum spicatum* var. *formosanum* (Honda) Ohwi | 1 | 1 | 1 |  |  | 1 | 1 | 1 |  |  |
| *Veratrum formosanum* O.Loes. |  |  |  |  |  | 1 | 1 | 1 |  |  |
| *Veronica morrisonicola* Hayata | 1 | 1 | 1 |  |  | 1 | 1 | 1 |  |  |
| *Viola adenothrix* Hayata var. *adenothrix* | 1 | 1 | 1 |  |  | 1 | 1 | 1 |  |  |
| *Yushania niitakayamensis* (Hayata) Keng f. | 1 | 1 | 1 |  |  | 1 | 1 | 1 |  |  |

^1^ The first (S1), second (S2), and the third (S3) survey. If the species was presence, it was marked as “1”.

^2^ Diff (1,2): the species difference between the first survey and the second survey; Diff (2,3): the species difference between the second survey and the third survey, “+” means newly recorded species, “−” means disappeared species.

**Table S5**. The correlation analysis between the species number variation and the mean annual temperature, the mean annual precipitation, or the altitude of the summit.

| **Survey** | **Variation of species number** | **Factor** | **T** | **Correlation coefficient** | ***p*-value** |
| --- | --- | --- | --- | --- | --- |
| S2 | increase | MAT^1^ | 0.16 | 0.08 | 0.88 |
|  |  | MAP^2^ | 0.16 | 0.08 | 0.88 |
|  |  | altitude | -0.08 | -0.04 | 0.94 |
|  | disappear | MAT | 1.11 | 0.49 | 0.33 |
|  |  | MAP | -0.69 | -0.32 | 0.53 |
|  |  | altitude | -2.13 | -0.73 | 0.10 |
| S3 | increase | MAT | 0.15 | 0.08 | 0.88 |
|  |  | MAP | -0.38 | -0.19 | 0.73 |
|  |  | altitude | -0.30 | -0.15 | 0.78 |
|  | disappear | MAT | 0.16 | 0.08 | 0.88 |
|  |  | MAP | -1.65 | -0.64 | 0.17 |
|  |  | altitude | 0.31 | 0.16 | 0.77 |

^1^ The mean annual temperature.

^2^ The mean annual precipitation.

**Table S6**. The generalized linear model result of the species cover change rate and climate niche.

| **Rate of cover change** | **Variable** | **Estimate** | **Std. Error** | ***t*-value** | ***p*-value** |
| --- | --- | --- | --- | --- | --- |
| S2/S1 | Intercept | 1.1066 | 0.5867 | 1.886 | 0.060 |
|  | T^1^ | 0.0216 | 0.0489 | 0.442 | 0.659 |
|  | P^2^ | -0.0001 | 0.0002 | -0.803 | 0.423 |
| S3/S2 | Intercept | 2.2828 | 0.8202 | 2.783 | 0.006 |
|  | T | -0.0201 | 0.0689 | -0.292 | 0.770 |
|  | P | -0.0009 | 0.0003 | -3.497 | 0.001 |

^1^Temperature niche.

^2^ Precipitation niche.

**Table S7**. The t-test results for the thermophilization and moist-philization indicator during the monitoring cycles at each summit.

| **Indicator** | **MC^1^** | **Summit** | **t** | **df** | ***p*-value** |
| --- | --- | --- | --- | --- | --- |
| Thermophilization | 2 | SEN | 1.694 | 7 | 0.134 |
|  | 2 | SUN | -3.565 | 7 | 0.009 |
|  | 2 | YAT | -3.435 | 7 | 0.011 |
|  | 2 | DSH | 0.415 | 7 | 0.690 |
|  | 2 | JNJ | 3.397 | 7 | 0.011 |
|  | 2 | TSW | 0.472 | 7 | 0.651 |
|  | 3 | SEN | -0.374 | 7 | 0.720 |
|  | 3 | SUN | 2.461 | 7 | 0.043 |
|  | 3 | YAT | -0.677 | 7 | 0.520 |
|  | 3 | DSH | 2.999 | 7 | 0.020 |
|  | 3 | JNJ | 1.165 | 7 | 0.282 |
|  | 3 | JNJ^2^ | 3.2995 | 6 | 0.016 |
|  | 3 | TSW | 1.161 | 7 | 0.284 |
| Moist-philization | 2 | SEN | 0.398 | 7 | 0.703 |
|  | 2 | SUN | -0.947 | 7 | 0.375 |
|  | 2 | YAT | -0.178 | 7 | 0.864 |
|  | 2 | DSH | 0.559 | 7 | 0.594 |
|  | 2 | JNJ | 0.583 | 7 | 0.578 |
|  | 2 | TSW | -1.463 | 7 | 0.187 |
|  | 3 | SEN | -2.511 | 7 | 0.040 |
|  | 3 | SUN | -3.783 | 7 | 0.007 |
|  | 3 | YAT | -6.133 | 7 | 0.000 |
|  | 3 | DSH | -2.527 | 7 | 0.039 |
|  | 3 | JNJ | -0.068 | 7 | 0.948 |
|  | 3 | TSW | -1.284 | 7 | 0.240 |

^1^ The monitoring cycle

^2^ The test result of excluded outliers.

**Table S8**. The occurrence data reference of 89 species from the Global Biodiversity Information Facility (GBIF, <URL:https://www.gbif.org>)

| **GBIF Key** | **DOI** | **License** | **Access date**  **(YYYY-mm-dd)** | **Total records** |
| --- | --- | --- | --- | --- |
| 0077525-210914110416597 | 10.15468/dl.ytsh6w | http://creativecommons.org/licenses/by-nc/4.0/legalcode | 2021-12-11 | 378 |
| 0077524-210914110416597 | 10.15468/dl.tb9469 | http://creativecommons.org/licenses/by-nc/4.0/legalcode | 2021-12-11 | 287 |
| 0077523-210914110416597 | 10.15468/dl.re6a8b | http://creativecommons.org/licenses/by-nc/4.0/legalcode | 2021-12-11 | 400 |
| 0077522-210914110416597 | 10.15468/dl.7sn3kc | http://creativecommons.org/licenses/by-nc/4.0/legalcode | 2021-12-11 | 174 |
| 0077521-210914110416597 | 10.15468/dl.kzm2xf | http://creativecommons.org/licenses/by-nc/4.0/legalcode | 2021-12-11 | 151 |
| 0077520-210914110416597 | 10.15468/dl.w4ecma | http://creativecommons.org/licenses/by-nc/4.0/legalcode | 2021-12-11 | 316 |
| 0077519-210914110416597 | 10.15468/dl.uhgh64 | http://creativecommons.org/licenses/by-nc/4.0/legalcode | 2021-12-11 | 469 |
| 0077518-210914110416597 | 10.15468/dl.bxqbum | http://creativecommons.org/licenses/by-nc/4.0/legalcode | 2021-12-11 | 44 |
| 0077517-210914110416597 | 10.15468/dl.sn629q | http://creativecommons.org/licenses/by-nc/4.0/legalcode | 2021-12-11 | 250 |
| 0077516-210914110416597 | 10.15468/dl.r4jy4y | http://creativecommons.org/licenses/by/4.0/legalcode | 2021-12-11 | 107 |
| 0077515-210914110416597 | 10.15468/dl.2sv4fx | http://creativecommons.org/licenses/by-nc/4.0/legalcode | 2021-12-11 | 53 |
| 0077514-210914110416597 | 10.15468/dl.vax4uv | http://creativecommons.org/licenses/by-nc/4.0/legalcode | 2021-12-11 | 64 |
| 0077513-210914110416597 | 10.15468/dl.eyejbf | http://creativecommons.org/licenses/by-nc/4.0/legalcode | 2021-12-11 | 293 |
| 0077512-210914110416597 | 10.15468/dl.7trsdp | http://creativecommons.org/licenses/by-nc/4.0/legalcode | 2021-12-11 | 136 |
| 0077511-210914110416597 | 10.15468/dl.duqy9s | http://creativecommons.org/licenses/by-nc/4.0/legalcode | 2021-12-11 | 26 |
| 0077509-210914110416597 | 10.15468/dl.k68pdh | http://creativecommons.org/licenses/by-nc/4.0/legalcode | 2021-12-11 | 264 |
| 0077508-210914110416597 | 10.15468/dl.z69pg5 | http://creativecommons.org/licenses/by-nc/4.0/legalcode | 2021-12-11 | 271 |
| 0077507-210914110416597 | 10.15468/dl.mgcqx2 | http://creativecommons.org/licenses/by-nc/4.0/legalcode | 2021-12-11 | 178 |
| 0077506-210914110416597 | 10.15468/dl.3uug2d | http://creativecommons.org/licenses/by-nc/4.0/legalcode | 2021-12-11 | 132 |
| 0077505-210914110416597 | 10.15468/dl.d6fpe6 | http://creativecommons.org/licenses/by-nc/4.0/legalcode | 2021-12-11 | 775 |
| 0077504-210914110416597 | 10.15468/dl.qx7dxw | http://creativecommons.org/licenses/by-nc/4.0/legalcode | 2021-12-11 | 88 |
| 0077502-210914110416597 | 10.15468/dl.3w4ndw | http://creativecommons.org/licenses/by-nc/4.0/legalcode | 2021-12-11 | 90 |
| 0077501-210914110416597 | 10.15468/dl.hn3n3m | http://creativecommons.org/licenses/by-nc/4.0/legalcode | 2021-12-11 | 2920 |
| 0077500-210914110416597 | 10.15468/dl.9nzpvm | http://creativecommons.org/licenses/by-nc/4.0/legalcode | 2021-12-11 | 225 |
| 0077499-210914110416597 | 10.15468/dl.azfpdv | http://creativecommons.org/licenses/by-nc/4.0/legalcode | 2021-12-11 | 571 |
| 0077498-210914110416597 | 10.15468/dl.s24zh5 | http://creativecommons.org/licenses/by-nc/4.0/legalcode | 2021-12-11 | 144 |
| 0077497-210914110416597 | 10.15468/dl.h3zvt4 | http://creativecommons.org/licenses/by-nc/4.0/legalcode | 2021-12-11 | 412 |
| 0077496-210914110416597 | 10.15468/dl.ud9h62 | http://creativecommons.org/licenses/by-nc/4.0/legalcode | 2021-12-11 | 123 |
| 0077494-210914110416597 | 10.15468/dl.qhmayb | http://creativecommons.org/licenses/by-nc/4.0/legalcode | 2021-12-11 | 50575 |
| 0077493-210914110416597 | 10.15468/dl.24vufg | http://creativecommons.org/licenses/by-nc/4.0/legalcode | 2021-12-11 | 254062 |
| 0077492-210914110416597 | 10.15468/dl.4w7suu | http://creativecommons.org/licenses/by-nc/4.0/legalcode | 2021-12-11 | 367 |
| 0077491-210914110416597 | 10.15468/dl.643jzv | http://creativecommons.org/licenses/by-nc/4.0/legalcode | 2021-12-11 | 6274 |
| 0077490-210914110416597 | 10.15468/dl.awq23u | http://creativecommons.org/licenses/by-nc/4.0/legalcode | 2021-12-11 | 180 |
| 0077489-210914110416597 | 10.15468/dl.vbjcsc | http://creativecommons.org/licenses/by-nc/4.0/legalcode | 2021-12-11 | 79441 |
| 0077488-210914110416597 | 10.15468/dl.z7gy3e | http://creativecommons.org/licenses/by-nc/4.0/legalcode | 2021-12-11 | 153 |
| 0077487-210914110416597 | 10.15468/dl.97ufk2 | http://creativecommons.org/licenses/by-nc/4.0/legalcode | 2021-12-11 | 346 |
| 0077486-210914110416597 | 10.15468/dl.dj43nw | http://creativecommons.org/licenses/by-nc/4.0/legalcode | 2021-12-11 | 230 |
| 0077485-210914110416597 | 10.15468/dl.n8aenk | http://creativecommons.org/licenses/by-nc/4.0/legalcode | 2021-12-11 | 1303 |
| 0077483-210914110416597 | 10.15468/dl.xp6tfn | http://creativecommons.org/licenses/by-nc/4.0/legalcode | 2021-12-11 | 100 |
| 0077482-210914110416597 | 10.15468/dl.s8u28d | http://creativecommons.org/publicdomain/zero/1.0/legalcode | 2021-12-11 | 136 |
| 0077481-210914110416597 | 10.15468/dl.uhgg65 | http://creativecommons.org/licenses/by-nc/4.0/legalcode | 2021-12-11 | 108 |
| 0077480-210914110416597 | 10.15468/dl.mme74w | http://creativecommons.org/licenses/by-nc/4.0/legalcode | 2021-12-11 | 2952 |
| 0077479-210914110416597 | 10.15468/dl.dukcr6 | http://creativecommons.org/licenses/by-nc/4.0/legalcode | 2021-12-11 | 41 |
| 0077478-210914110416597 | 10.15468/dl.48mgp6 | http://creativecommons.org/licenses/by-nc/4.0/legalcode | 2021-12-11 | 45 |
| 0077477-210914110416597 | 10.15468/dl.pvvwzx | http://creativecommons.org/licenses/by-nc/4.0/legalcode | 2021-12-11 | 37 |
| 0077476-210914110416597 | 10.15468/dl.rrsc6m | http://creativecommons.org/licenses/by-nc/4.0/legalcode | 2021-12-11 | 271 |
| 0077475-210914110416597 | 10.15468/dl.ujg3pj | http://creativecommons.org/licenses/by-nc/4.0/legalcode | 2021-12-11 | 343 |
| 0077472-210914110416597 | 10.15468/dl.3ahqdd | http://creativecommons.org/licenses/by-nc/4.0/legalcode | 2021-12-11 | 101 |
| 0077471-210914110416597 | 10.15468/dl.4ek2gu | http://creativecommons.org/licenses/by-nc/4.0/legalcode | 2021-12-11 | 203 |
| 0077470-210914110416597 | 10.15468/dl.yweef9 | http://creativecommons.org/licenses/by-nc/4.0/legalcode | 2021-12-11 | 363 |
| 0077469-210914110416597 | 10.15468/dl.kf2ghp | http://creativecommons.org/licenses/by-nc/4.0/legalcode | 2021-12-11 | 413 |
| 0077467-210914110416597 | 10.15468/dl.gh5kms | http://creativecommons.org/licenses/by-nc/4.0/legalcode | 2021-12-11 | 220 |
| 0077466-210914110416597 | 10.15468/dl.ybveuw | http://creativecommons.org/licenses/by-nc/4.0/legalcode | 2021-12-11 | 922697 |
| 0077464-210914110416597 | 10.15468/dl.qz4h9q | http://creativecommons.org/licenses/by-nc/4.0/legalcode | 2021-12-11 | 276176 |
| 0077463-210914110416597 | 10.15468/dl.38wwwp | http://creativecommons.org/licenses/by-nc/4.0/legalcode | 2021-12-11 | 163 |
| 0077460-210914110416597 | 10.15468/dl.89wqdz | http://creativecommons.org/licenses/by-nc/4.0/legalcode | 2021-12-11 | 329 |
| 0077459-210914110416597 | 10.15468/dl.vnqjtu | http://creativecommons.org/licenses/by-nc/4.0/legalcode | 2021-12-11 | 164 |
| 0077458-210914110416597 | 10.15468/dl.p9sn6f | http://creativecommons.org/licenses/by-nc/4.0/legalcode | 2021-12-11 | 42930 |
| 0077457-210914110416597 | 10.15468/dl.9jfp2b | http://creativecommons.org/licenses/by-nc/4.0/legalcode | 2021-12-11 | 113 |
| 0077456-210914110416597 | 10.15468/dl.67aubb | http://creativecommons.org/licenses/by-nc/4.0/legalcode | 2021-12-11 | 133 |
| 0077455-210914110416597 | 10.15468/dl.rywubj | http://creativecommons.org/licenses/by-nc/4.0/legalcode | 2021-12-11 | 111 |
| 0077454-210914110416597 | 10.15468/dl.ctvetb | http://creativecommons.org/licenses/by-nc/4.0/legalcode | 2021-12-11 | 260 |
| 0077453-210914110416597 | 10.15468/dl.nh7dq6 | http://creativecommons.org/licenses/by-nc/4.0/legalcode | 2021-12-11 | 8450 |
| 0077452-210914110416597 | 10.15468/dl.keqayc | http://creativecommons.org/licenses/by-nc/4.0/legalcode | 2021-12-11 | 145 |
| 0077451-210914110416597 | 10.15468/dl.drw8eq | http://creativecommons.org/licenses/by-nc/4.0/legalcode | 2021-12-11 | 2070 |
| 0077450-210914110416597 | 10.15468/dl.j8pfrz | http://creativecommons.org/licenses/by-nc/4.0/legalcode | 2021-12-11 | 105 |
| 0077449-210914110416597 | 10.15468/dl.w5bxaz | http://creativecommons.org/licenses/by-nc/4.0/legalcode | 2021-12-11 | 141 |
| 0077448-210914110416597 | 10.15468/dl.x9rbmu | http://creativecommons.org/licenses/by-nc/4.0/legalcode | 2021-12-11 | 278 |
| 0077445-210914110416597 | 10.15468/dl.dpqmys | http://creativecommons.org/licenses/by-nc/4.0/legalcode | 2021-12-11 | 1161 |
| 0077444-210914110416597 | 10.15468/dl.9ccz7n | http://creativecommons.org/licenses/by-nc/4.0/legalcode | 2021-12-11 | 23 |
| 0077443-210914110416597 | 10.15468/dl.dvtqsc | http://creativecommons.org/licenses/by-nc/4.0/legalcode | 2021-12-11 | 556 |
| 0077442-210914110416597 | 10.15468/dl.rjzx37 | http://creativecommons.org/licenses/by-nc/4.0/legalcode | 2021-12-11 | 195 |
| 0077441-210914110416597 | 10.15468/dl.jx4njn | http://creativecommons.org/licenses/by-nc/4.0/legalcode | 2021-12-11 | 18593 |
| 0077440-210914110416597 | 10.15468/dl.dxhkgq | http://creativecommons.org/licenses/by-nc/4.0/legalcode | 2021-12-11 | 57 |
| 0077439-210914110416597 | 10.15468/dl.d3atpy | http://creativecommons.org/publicdomain/zero/1.0/legalcode | 2021-12-11 | 40 |
| 0077438-210914110416597 | 10.15468/dl.bjb6ry | http://creativecommons.org/licenses/by-nc/4.0/legalcode | 2021-12-11 | 80 |
| 0077437-210914110416597 | 10.15468/dl.gkb6sk | http://creativecommons.org/licenses/by/4.0/legalcode | 2021-12-11 | 25 |
| 0077436-210914110416597 | 10.15468/dl.qk4wzs | http://creativecommons.org/licenses/by-nc/4.0/legalcode | 2021-12-11 | 296 |
| 0077435-210914110416597 | 10.15468/dl.btnc9a | http://creativecommons.org/licenses/by-nc/4.0/legalcode | 2021-12-11 | 393 |
| 0077434-210914110416597 | 10.15468/dl.ee2uay | http://creativecommons.org/licenses/by-nc/4.0/legalcode | 2021-12-11 | 83 |
| 0077433-210914110416597 | 10.15468/dl.z7pcra | http://creativecommons.org/licenses/by-nc/4.0/legalcode | 2021-12-11 | 129 |
| 0077432-210914110416597 | 10.15468/dl.7uc5t5 | http://creativecommons.org/licenses/by-nc/4.0/legalcode | 2021-12-11 | 7 |
| 0077431-210914110416597 | 10.15468/dl.tcczn7 | http://creativecommons.org/licenses/by-nc/4.0/legalcode | 2021-12-11 | 1516 |
| 0077430-210914110416597 | 10.15468/dl.deuywc | http://creativecommons.org/licenses/by-nc/4.0/legalcode | 2021-12-11 | 1496 |
| 0077429-210914110416597 | 10.15468/dl.g4cm4u | http://creativecommons.org/licenses/by-nc/4.0/legalcode | 2021-12-11 | 560 |
| 0077428-210914110416597 | 10.15468/dl.7qmddv | http://creativecommons.org/licenses/by-nc/4.0/legalcode | 2021-12-11 | 30 |
| 0077427-210914110416597 | 10.15468/dl.knppd2 | http://creativecommons.org/licenses/by/4.0/legalcode | 2021-12-11 | 49 |
| 0077426-210914110416597 | 10.15468/dl.tgdjyp | http://creativecommons.org/licenses/by-nc/4.0/legalcode | 2021-12-11 | 673 |
| 0077425-210914110416597 | 10.15468/dl.g87ugu | http://creativecommons.org/licenses/by-nc/4.0/legalcode | 2021-12-11 | 212 |

**Table S9**. The top cover surface type of all section plots in six summits of each survey

| Survey | Region | Summit | Section^1^ | Top cover surface type (%) | | | | | | |
| --- | --- | --- | --- | --- | --- | --- | --- | --- | --- | --- |
|  |  |  |  | Vascular  plants | Solid  rock | Scree | Lichens | Bryophytes | Bare  ground | Litter |
| 1st | DAS | SUN | E05 | 72.5 | 18.0 | 5.0 | 2.0 | 1.0 | 0.5 | 1.0 |
| 1st | DAS | SUN | E10 | 59.0 | 12.0 | 18.0 | 3.0 | 1.0 | 1.0 | 6.0 |
| 1st | DAS | SUN | N05 | 74.5 | 12.0 | 8.0 | 2.0 | 2.0 | 1.0 | 0.5 |
| 1st | DAS | SUN | N10 | 87.5 | 5.0 | 3.0 | 2.0 | 1.0 | 1.0 | 0.5 |
| 1st | DAS | SUN | S05 | 45.7 | 24.0 | 10.0 | 1.5 | 0.8 | 2.0 | 6.0 |
| 1st | DAS | SUN | S10 | 58.2 | 23.0 | 7.0 | 1.2 | 2.4 | 2.4 | 7.0 |
| 1st | DAS | SUN | W05 | 78.0 | 10.0 | 5.0 | 1.0 | 2.0 | 3.0 | 1.0 |
| 1st | DAS | SUN | W10 | 85.0 | 5.0 | 3.0 | 2.0 | 1.0 | 2.0 | 2.0 |
| 1st | DAS | YAT | E05 | 88.0 | 0.0 | 2.0 | 3.0 | 1.0 | 4.0 | 2.0 |
| 1st | DAS | YAT | E10 | 91.7 | 0.1 | 4.0 | 1.2 | 0.4 | 3.0 | 0.7 |
| 1st | DAS | YAT | N05 | 80.2 | 6.0 | 10.0 | 2.5 | 0.7 | 0.5 | 0.1 |
| 1st | DAS | YAT | N10 | 78.8 | 13.0 | 4.0 | 2.0 | 0.8 | 0.8 | 0.6 |
| 1st | DAS | YAT | S05 | 91.0 | 1.0 | 2.0 | 1.0 | 1.0 | 3.0 | 1.0 |
| 1st | DAS | YAT | S10 | 90.5 | 1.0 | 3.0 | 1.0 | 0.5 | 3.0 | 1.0 |
| 1st | DAS | YAT | W05 | 78.0 | 10.0 | 9.0 | 1.0 | 1.5 | 0.0 | 0.5 |
| 1st | DAS | YAT | W10 | 47.3 | 30.0 | 22.0 | 0.1 | 0.5 | 0.0 | 0.1 |
| 1st | DAS | SEN | E05 | 75.0 | 13.0 | 8.0 | 0.3 | 1.0 | 2.0 | 0.7 |
| 1st | DAS | SEN | E10 | 77.5 | 10.0 | 6.0 | 1.0 | 0.5 | 3.0 | 2.0 |
| 1st | DAS | SEN | N05 | 53.0 | 11.0 | 25.0 | 1.0 | 6.0 | 2.0 | 2.0 |
| 1st | DAS | SEN | N10 | 63.3 | 15.0 | 11.0 | 2.5 | 4.5 | 3.0 | 0.7 |
| 1st | DAS | SEN | S05 | 42.5 | 25.0 | 15.0 | 2.0 | 0.5 | 9.0 | 6.0 |
| 1st | DAS | SEN | S10 | 30.0 | 50.7 | 15.0 | 1.0 | 0.3 | 2.0 | 1.0 |
| 1st | DAS | SEN | W05 | 84.0 | 3.5 | 7.5 | 1.3 | 0.2 | 3.0 | 0.5 |
| 1st | DAS | SEN | W10 | 70.0 | 15.0 | 7.0 | 1.5 | 0.5 | 3.5 | 2.5 |
| 1st | SYU | JNJ | E05 | 73.0 | 1.2 | 23.2 | 0.2 | 0.0 | 0.3 | 2.4 |
| 1st | SYU | JNJ | E10 | 75.0 | 9.5 | 13.9 | 0.7 | 0.0 | 0.8 | 0.4 |
| 1st | SYU | JNJ | N05 | 90.0 | 0.0 | 0.2 | 3.6 | 0.2 | 0.2 | 6.6 |
| 1st | SYU | JNJ | N10 | 92.0 | 0.0 | 1.0 | 1.4 | 0.1 | 0.1 | 5.6 |
| 1st | SYU | JNJ | S05 | 57.0 | 8.7 | 25.2 | 0.0 | 0.0 | 3.5 | 6.1 |
| 1st | SYU | JNJ | S10 | 81.0 | 12.0 | 5.6 | 0.0 | 0.1 | 0.8 | 1.3 |
| 1st | SYU | JNJ | W05 | 79.4 | 2.4 | 5.6 | 0.0 | 0.8 | 3.2 | 8.7 |
| 1st | SYU | JNJ | W10 | 84.2 | 7.9 | 2.6 | 0.4 | 0.4 | 0.0 | 5.2 |
| 1st | SYU | DSH | E05 | 81.5 | 3.0 | 3.5 | 1.5 | 0.5 | 0.0 | 10.0 |
| 1st | SYU | DSH | E10 | 84.0 | 3.0 | 5.0 | 2.0 | 0.5 | 0.0 | 6.0 |
| 1st | SYU | DSH | N05 | 75.0 | 5.0 | 8.0 | 4.0 | 0.0 | 1.0 | 7.0 |
| 1st | SYU | DSH | N10 | 79.0 | 2.5 | 7.0 | 2.0 | 0.5 | 0.3 | 9.0 |
| 1st | SYU | DSH | S05 | 72.5 | 5.0 | 15.0 | 0.5 | 2.0 | 2.0 | 3.0 |
| 1st | SYU | DSH | S10 | 57.0 | 15.0 | 25.0 | 0.0 | 0.0 | 0.5 | 3.0 |
| 1st | SYU | DSH | W05 | 60.0 | 12.0 | 18.0 | 2.0 | 0.1 | 0.5 | 7.4 |
| 1st | SYU | DSH | W10 | 80.0 | 6.0 | 4.0 | 2.0 | 0.5 | 0.5 | 7.0 |
| 1st | SYU | TSW | E05 | 80.5 | 2.6 | 8.5 | 1.1 | 0.4 | 2.6 | 4.4 |
| 1st | SYU | TSW | E10 | 69.3 | 9.8 | 7.6 | 0.4 | 0.8 | 0.3 | 11.9 |
| 1st | SYU | TSW | N05 | 70.3 | 0.7 | 0.6 | 20.3 | 2.0 | 0.0 | 6.1 |
| 1st | SYU | TSW | N10 | 89.0 | 1.1 | 1.4 | 4.1 | 2.5 | 0.0 | 5.8 |
| 1st | SYU | TSW | S05 | 70.5 | 1.9 | 14.1 | 0.0 | 2.3 | 1.4 | 9.9 |
| 1st | SYU | TSW | S10 | 63.0 | 15.8 | 8.1 | 0.0 | 3.8 | 0.9 | 8.5 |
| 1st | SYU | TSW | W05 | 82.0 | 0.0 | 10.0 | 0.0 | 1.2 | 0.0 | 7.5 |
| 1st | SYU | TSW | W10 | 77.5 | 1.3 | 14.1 | 2.7 | 2.0 | 0.0 | 3.4 |
| 2nd | DAS | SUN | E05 | 85.0 | 5.0 | 5.0 | 1.0 | 0.5 | 3.0 | 0.5 |
| 2nd | DAS | SUN | E10 | 82.0 | 7.0 | 9.0 | 0.2 | 0.2 | 0.2 | 1.4 |
| 2nd | DAS | SUN | S05 | 75.0 | 10.0 | 12.0 | 0.5 | 1.5 | 0.5 | 0.5 |
| 2nd | DAS | SUN | S10 | 74.0 | 12.0 | 10.0 | 0.5 | 0.5 | 2.0 | 1.0 |
| 2nd | DAS | SUN | W05 | 69.0 | 15.0 | 8.0 | 2.0 | 3.0 | 1.0 | 2.0 |
| 2nd | DAS | SUN | W10 | 77.0 | 10.0 | 5.0 | 2.0 | 1.0 | 2.0 | 3.0 |
| 2nd | DAS | SUN | N05 | 70.0 | 15.0 | 8.0 | 1.0 | 0.5 | 2.5 | 3.0 |
| 2nd | DAS | SUN | N10 | 87.0 | 9.0 | 2.0 | 0.8 | 0.2 | 0.2 | 0.8 |
| 2nd | DAS | YAT | E05 | 92.0 | 2.0 | 2.0 | 0.5 | 0.5 | 2.0 | 1.0 |
| 2nd | DAS | YAT | E10 | 94.5 | 1.0 | 1.0 | 1.0 | 0.5 | 1.0 | 1.0 |
| 2nd | DAS | YAT | S05 | 85.8 | 3.0 | 8.0 | 1.0 | 0.5 | 1.5 | 0.2 |
| 2nd | DAS | YAT | S10 | 92.0 | 0.5 | 3.0 | 1.0 | 0.5 | 1.0 | 2.0 |
| 2nd | DAS | YAT | W05 | 69.0 | 17.0 | 12.0 | 0.6 | 1.0 | 0.2 | 0.2 |
| 2nd | DAS | YAT | W10 | 18.0 | 70.0 | 11.0 | 0.1 | 0.8 | 0.0 | 0.1 |
| 2nd | DAS | YAT | N05 | 85.0 | 7.0 | 4.0 | 0.5 | 2.0 | 0.5 | 1.0 |
| 2nd | DAS | YAT | N10 | 73.0 | 15.0 | 10.0 | 0.1 | 1.0 | 0.1 | 0.8 |
| 2nd | DAS | SEN | E05 | 76.0 | 15.0 | 5.0 | 0.2 | 1.0 | 0.5 | 2.3 |
| 2nd | DAS | SEN | E10 | 78.0 | 12.0 | 8.0 | 0.2 | 0.5 | 0.3 | 1.0 |
| 2nd | DAS | SEN | S05 | 69.0 | 10.0 | 17.0 | 2.0 | 0.5 | 1.5 | 1.5 |
| 2nd | DAS | SEN | S10 | 18.0 | 60.0 | 20.0 | 0.5 | 0.2 | 0.3 | 1.0 |
| 2nd | DAS | SEN | W05 | 75.0 | 11.0 | 8.0 | 2.0 | 1.0 | 2.0 | 1.0 |
| 2nd | DAS | SEN | W10 | 65.0 | 16.0 | 10.0 | 3.0 | 3.0 | 1.0 | 2.0 |
| 2nd | DAS | SEN | N05 | 63.0 | 5.0 | 20.0 | 1.0 | 1.0 | 5.0 | 5.0 |
| 2nd | DAS | SEN | N10 | 64.0 | 6.0 | 23.0 | 1.0 | 1.0 | 3.0 | 2.0 |
| 2nd | SYU | JNJ | E05 | 71.0 | 1.5 | 20.5 | 0.5 | 0.5 | 4.0 | 2.0 |
| 2nd | SYU | JNJ | E10 | 71.9 | 10.0 | 12.0 | 0.1 | 0.1 | 4.0 | 2.0 |
| 2nd | SYU | JNJ | S05 | 63.0 | 14.0 | 17.0 | 0.0 | 0.8 | 0.5 | 4.7 |
| 2nd | SYU | JNJ | S10 | 58.2 | 25.0 | 8.0 | 0.0 | 0.4 | 0.9 | 7.5 |
| 2nd | SYU | JNJ | W05 | 85.0 | 4.0 | 5.1 | 0.5 | 0.4 | 2.0 | 3.0 |
| 2nd | SYU | JNJ | W10 | 85.0 | 7.0 | 2.0 | 1.0 | 0.5 | 0.5 | 4.0 |
| 2nd | SYU | JNJ | N05 | 94.7 | 0.2 | 1.5 | 0.8 | 0.2 | 0.1 | 2.5 |
| 2nd | SYU | JNJ | N10 | 92.0 | 0.1 | 0.2 | 2.5 | 0.1 | 0.1 | 5.0 |
| 2nd | SYU | DSH | E05 | 84.0 | 3.0 | 3.0 | 1.0 | 0.0 | 1.0 | 8.0 |
| 2nd | SYU | DSH | E10 | 84.0 | 3.0 | 6.0 | 2.5 | 1.0 | 0.5 | 3.0 |
| 2nd | SYU | DSH | S05 | 55.3 | 3.5 | 25.0 | 1.5 | 0.8 | 0.9 | 13.0 |
| 2nd | SYU | DSH | S10 | 56.5 | 15.0 | 23.0 | 0.5 | 1.0 | 1.0 | 3.0 |
| 2nd | SYU | DSH | W05 | 61.4 | 17.0 | 15.0 | 1.0 | 0.5 | 0.1 | 5.0 |
| 2nd | SYU | DSH | W10 | 87.0 | 8.0 | 2.0 | 0.5 | 1.0 | 0.5 | 1.0 |
| 2nd | SYU | DSH | N05 | 77.5 | 5.0 | 8.0 | 2.0 | 0.5 | 2.0 | 5.0 |
| 2nd | SYU | DSH | N10 | 78.5 | 5.0 | 5.0 | 2.0 | 1.0 | 0.5 | 8.0 |
| 2nd | SYU | TSW | E05 | 78.0 | 5.0 | 8.0 | 1.0 | 0.1 | 2.4 | 5.5 |
| 2nd | SYU | TSW | E10 | 62.7 | 17.0 | 12.0 | 0.0 | 0.1 | 0.2 | 8.0 |
| 2nd | SYU | TSW | S05 | 66.4 | 2.0 | 18.0 | 0.1 | 0.8 | 0.7 | 12.0 |
| 2nd | SYU | TSW | S10 | 60.7 | 16.0 | 10.0 | 0.0 | 0.2 | 0.1 | 13.0 |
| 2nd | SYU | TSW | W05 | 84.0 | 0.0 | 8.0 | 0.8 | 0.2 | 0.0 | 7.0 |
| 2nd | SYU | TSW | W10 | 80.0 | 1.0 | 10.0 | 3.0 | 3.0 | 1.0 | 2.0 |
| 2nd | SYU | TSW | N05 | 73.0 | 1.0 | 6.0 | 10.0 | 2.0 | 2.0 | 6.0 |
| 2nd | SYU | TSW | N10 | 92.0 | 0.0 | 3.0 | 1.8 | 0.2 | 1.0 | 2.0 |
| 3rd | DAS | SUN | E05 | 75.0 | 5.0 | 10.0 | 1.0 | 1.0 | 3.0 | 5.0 |
| 3rd | DAS | SUN | E10 | 65.0 | 10.0 | 20.0 | 0.5 | 0.0 | 9.0 | 0.5 |
| 3rd | DAS | SUN | S05 | 40.0 | 20.0 | 20.0 | 2.0 | 0.0 | 3.0 | 5.0 |
| 3rd | DAS | SUN | S10 | 50.0 | 15.0 | 20.0 | 0.0 | 3.0 | 5.0 | 7.0 |
| 3rd | DAS | SUN | W05 | 65.0 | 20.0 | 10.0 | 3.0 | 0.0 | 2.0 | 0.0 |
| 3rd | DAS | SUN | W10 | 64.0 | 15.0 | 10.0 | 0.5 | 0.0 | 10.0 | 0.5 |
| 3rd | DAS | SUN | N05 | 50.0 | 30.0 | 15.0 | 2.0 | 0.0 | 3.0 | 5.0 |
| 3rd | DAS | SUN | N10 | 70.0 | 10.0 | 10.0 | 3.0 | 0.0 | 2.0 | 5.0 |
| 3rd | DAS | YAT | E05 | 80.0 | 0.0 | 1.0 | 1.0 | 1.0 | 7.0 | 10.0 |
| 3rd | DAS | YAT | E10 | 87.0 | 0.5 | 2.5 | 1.5 | 0.0 | 3.5 | 5.0 |
| 3rd | DAS | YAT | S05 | 60.0 | 0.0 | 10.0 | 0.0 | 0.0 | 30.0 | 0.0 |
| 3rd | DAS | YAT | S10 | 75.0 | 0.0 | 5.0 | 3.0 | 0.0 | 17.0 | 0.0 |
| 3rd | DAS | YAT | W05 | 70.0 | 15.0 | 10.0 | 0.0 | 1.0 | 3.0 | 1.0 |
| 3rd | DAS | YAT | W10 | 40.0 | 40.0 | 11.0 | 1.5 | 2.5 | 2.0 | 3.0 |
| 3rd | DAS | YAT | N05 | 80.0 | 5.0 | 5.0 | 1.5 | 1.5 | 3.0 | 4.0 |
| 3rd | DAS | YAT | N10 | 70.0 | 15.0 | 5.0 | 0.5 | 0.5 | 4.0 | 5.0 |
| 3rd | DAS | SEN | E05 | 80.0 | 12.0 | 1.0 | 0.5 | 3.5 | 0.0 | 3.0 |
| 3rd | DAS | SEN | E10 | 65.0 | 10.0 | 10.0 | 2.0 | 8.0 | 3.0 | 2.0 |
| 3rd | DAS | SEN | S05 | 65.0 | 10.0 | 20.0 | 2.0 | 0.0 | 3.0 | 0.0 |
| 3rd | DAS | SEN | S10 | 40.0 | 20.0 | 26.0 | 2.0 | 2.0 | 0.0 | 10.0 |
| 3rd | DAS | SEN | W05 | 50.0 | 15.0 | 25.0 | 1.0 | 0.0 | 8.0 | 1.0 |
| 3rd | DAS | SEN | W10 | 45.0 | 20.0 | 15.0 | 2.0 | 3.0 | 10.0 | 5.0 |
| 3rd | DAS | SEN | N05 | 65.0 | 5.0 | 15.0 | 3.0 | 0.0 | 6.0 | 6.0 |
| 3rd | DAS | SEN | N10 | 75.0 | 5.0 | 10.0 | 1.0 | 0.0 | 5.0 | 4.0 |
| 3rd | SYU | JNJ | E05 | 65.0 | 1.5 | 20.0 | 0.1 | 0.1 | 5.0 | 8.3 |
| 3rd | SYU | JNJ | E10 | 70.0 | 10.0 | 12.0 | 0.1 | 0.1 | 5.0 | 2.8 |
| 3rd | SYU | JNJ | S05 | 60.0 | 14.0 | 20.0 | 0.1 | 0.1 | 2.4 | 3.5 |
| 3rd | SYU | JNJ | S10 | 60.0 | 25.0 | 8.0 | 0.1 | 0.4 | 1.0 | 5.6 |
| 3rd | SYU | JNJ | W05 | 85.0 | 3.0 | 5.0 | 1.0 | 0.5 | 2.0 | 3.5 |
| 3rd | SYU | JNJ | W10 | 85.0 | 6.0 | 3.0 | 1.0 | 0.5 | 0.5 | 4.0 |
| 3rd | SYU | JNJ | N05 | 95.0 | 0.2 | 1.0 | 0.8 | 0.1 | 0.2 | 2.7 |
| 3rd | SYU | JNJ | N10 | 90.0 | 0.1 | 0.1 | 5.0 | 0.1 | 1.0 | 3.7 |
| 3rd | SYU | DSH | E05 | 90.0 | 3.0 | 4.0 | 0.0 | 0.0 | 3.0 | 0.0 |
| 3rd | SYU | DSH | E10 | 82.8 | 3.3 | 12.0 | 0.7 | 0.0 | 0.2 | 1.0 |
| 3rd | SYU | DSH | S05 | 60.0 | 4.0 | 20.0 | 0.0 | 0.2 | 5.0 | 10.8 |
| 3rd | SYU | DSH | S10 | 60.0 | 15.0 | 20.0 | 1.0 | 2.0 | 0.0 | 2.0 |
| 3rd | SYU | DSH | W05 | 67.0 | 17.0 | 15.0 | 0.0 | 0.0 | 1.0 | 0.0 |
| 3rd | SYU | DSH | W10 | 87.0 | 5.0 | 5.0 | 0.5 | 0.5 | 1.0 | 1.0 |
| 3rd | SYU | DSH | N05 | 86.0 | 5.0 | 2.0 | 2.0 | 0.0 | 2.5 | 2.5 |
| 3rd | SYU | DSH | N10 | 78.0 | 5.0 | 7.0 | 1.0 | 0.0 | 4.0 | 6.0 |
| 3rd | SYU | TSW | E05 | 77.5 | 5.0 | 8.0 | 1.5 | 0.5 | 2.5 | 5.0 |
| 3rd | SYU | TSW | E10 | 63.0 | 17.0 | 10.0 | 2.0 | 1.0 | 1.0 | 5.0 |
| 3rd | SYU | TSW | S05 | 80.0 | 1.0 | 13.0 | 0.5 | 0.1 | 0.4 | 5.0 |
| 3rd | SYU | TSW | S10 | 65.0 | 20.0 | 8.0 | 0.1 | 0.5 | 0.9 | 5.5 |
| 3rd | SYU | TSW | W05 | 85.0 | 0.0 | 5.0 | 1.0 | 4.0 | 4.0 | 1.0 |
| 3rd | SYU | TSW | W10 | 84.0 | 0.0 | 3.0 | 3.0 | 3.0 | 6.0 | 1.0 |
| 3rd | SYU | TSW | N05 | 87.0 | 0.0 | 4.0 | 3.0 | 3.0 | 1.0 | 2.0 |
| 3rd | SYU | TSW | N10 | 91.0 | 1.0 | 1.0 | 1.0 | 2.0 | 2.0 | 2.0 |

1 The first letter of section indicates the cardinal direction of east (E), north (N), south (S), and west (W); the latter two digits indicates the vertical contours of 5-m and 10-m.

**
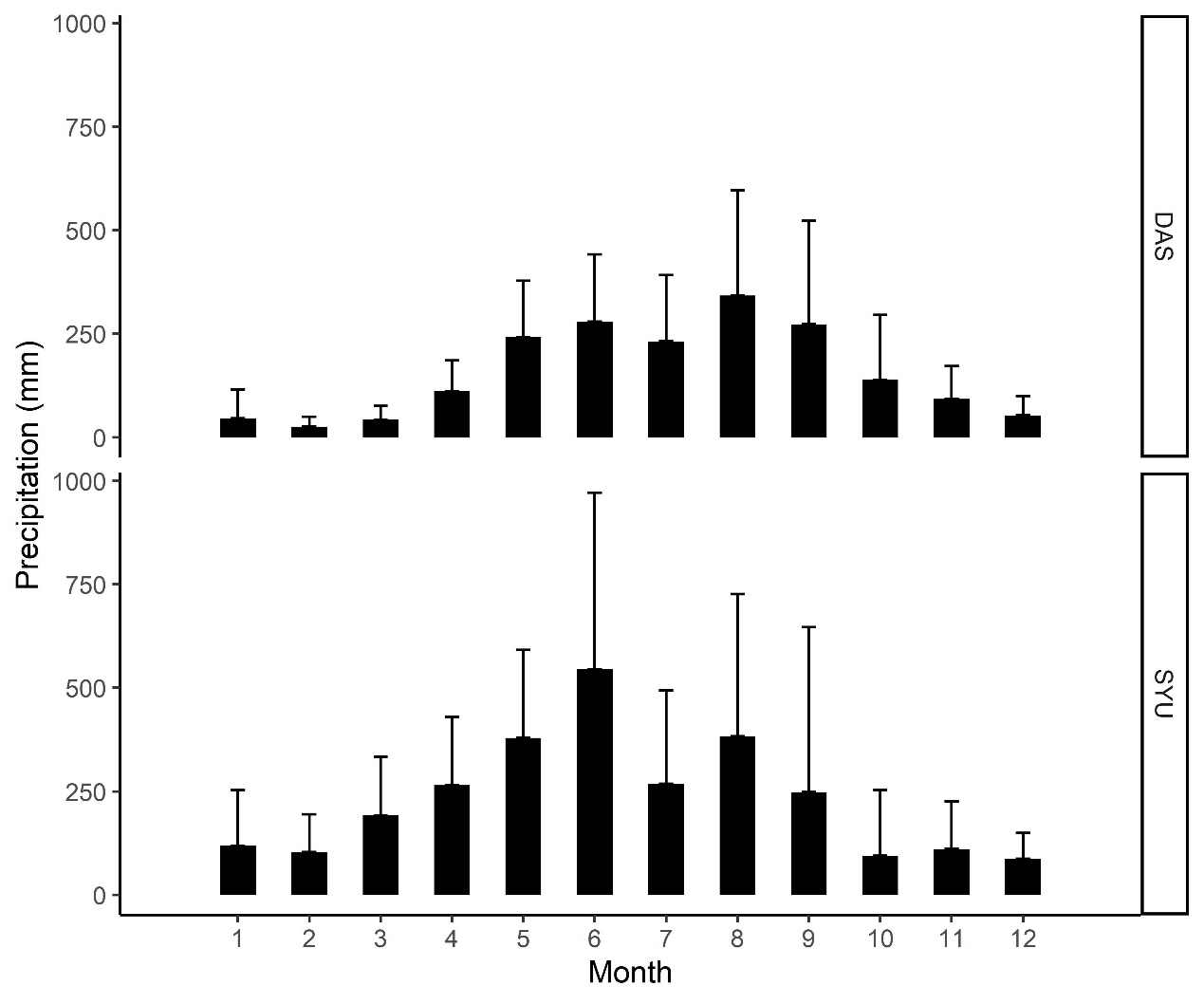
Figure S1.** The long-term (2005–2019) mean monthly precipitation of the DAS region and the SYU region.

**
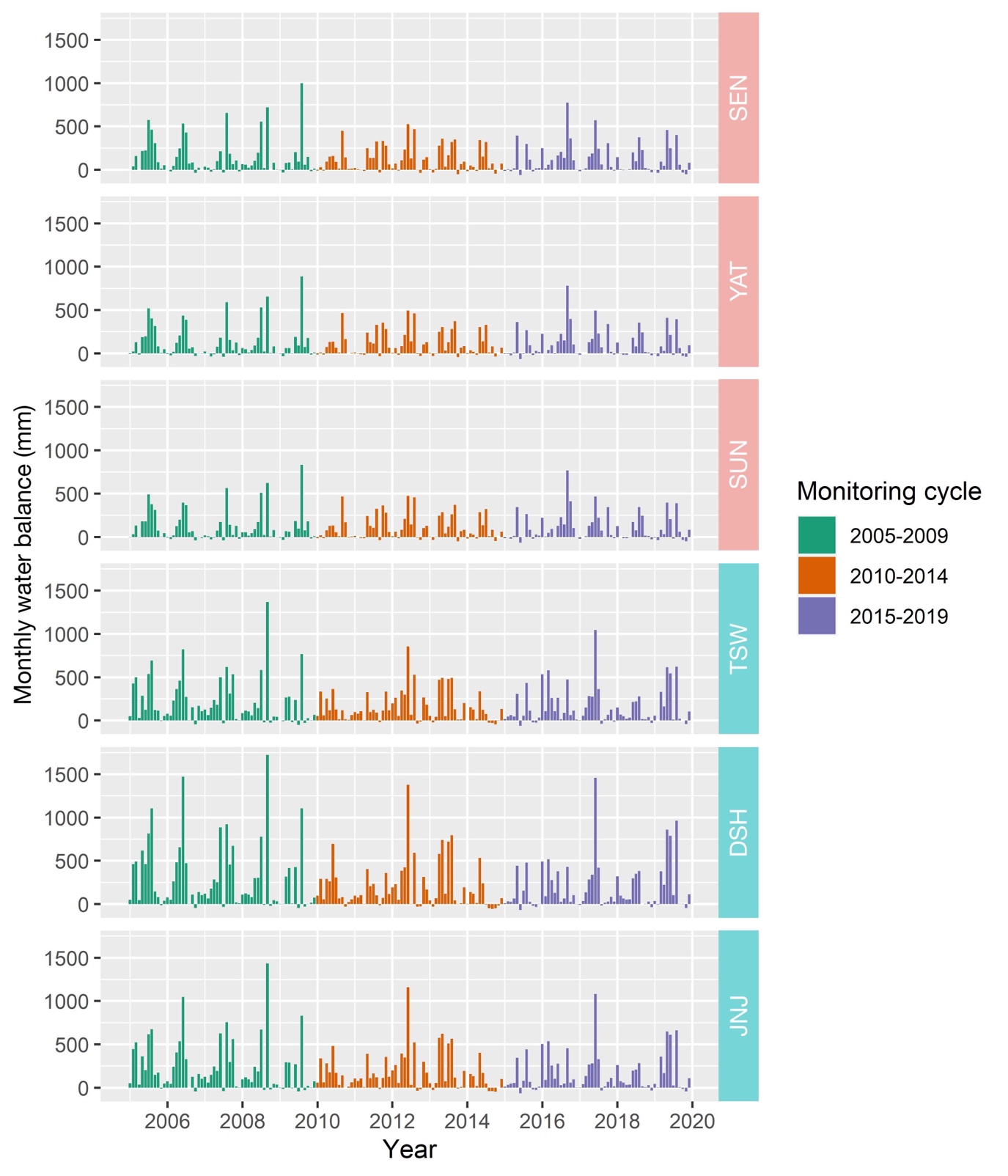
Figure S2**. The monthly water balance at each summit during the monitoring cycles.
